# Adaptive cognition implemented with a context-aware and flexible neuron for next-generation artificial intelligence

**DOI:** 10.1093/pnasnexus/pgac206

**Published:** 2022-09-29

**Authors:** Priyamvada Jadaun, Can Cui, Sam Liu, Jean Anne C Incorvia

**Affiliations:** Department of Electrical and Computer Engineering, The University of Texas at Austin, Austin, TX 78712, USA; Department of Electrical and Computer Engineering, The University of Texas at Austin, Austin, TX 78712, USA; Department of Electrical and Computer Engineering, The University of Texas at Austin, Austin, TX 78712, USA; Department of Electrical and Computer Engineering, The University of Texas at Austin, Austin, TX 78712, USA; Microelectronics Research Center, The University of Texas at Austin, Austin, TX 78758, USA

## Abstract

Neuromorphic computing mimics the organizational principles of the brain in its quest to replicate the brain’s intellectual abilities. An impressive ability of the brain is its adaptive intelligence, which allows the brain to regulate its functions “on the fly” to cope with myriad and ever-changing situations. In particular, the brain displays three adaptive and advanced intelligence abilities of context-awareness, cross frequency coupling, and feature binding. To mimic these adaptive cognitive abilities, we design and simulate a novel, hardware-based adaptive oscillatory neuron using a lattice of magnetic skyrmions. Charge current fed to the neuron reconfigures the skyrmion lattice, thereby modulating the neuron’s state, its dynamics and its transfer function “on the fly.” This adaptive neuron is used to demonstrate the three cognitive abilities, of which context-awareness and cross-frequency coupling have not been previously realized in hardware neurons. Additionally, the neuron is used to construct an adaptive artificial neural network (ANN) and perform context-aware diagnosis of breast cancer. Simulations show that the adaptive ANN diagnoses cancer with higher accuracy while learning faster and using a more compact and energy-efficient network than a nonadaptive ANN. The work further describes how hardware-based adaptive neurons can mitigate several critical challenges facing contemporary ANNs. Modern ANNs require large amounts of training data, energy, and chip area, and are highly task-specific; conversely, hardware-based ANNs built with adaptive neurons show faster learning, compact architectures, energy-efficiency, fault-tolerance, and can lead to the realization of broader artificial intelligence.

Significance Statement Among the many remarkable abilities of the brain, one that stands out is its sophisticated and adaptive intelligence, which allows it to successfully navigate dynamically changing situations, environments, goals, and contexts. However, state-of-the-art neuromorphic devices do not display such nimble information processing. Here, we report the design and simulation of an adaptive artificial neuron constructed from a lattice of magnetic textures called skyrmions. The skyrmion lattice being a smart material, can alter its structure and properties to suit dynamically changing situations. This adaptive neuron realizes three adaptive high-level intelligence capabilities, i.e. context-awareness, cross-frequency coupling, and feature binding. Additionally, adaptive neurons can mitigate critical challenges facing contemporary neural networks by enabling faster learning, compact architectures, broader artificial intelligence, energy-efficiency, and fault-tolerance.

## Introduction

Neuromorphic computing mimics the structural and functional principles of the human brain in hardware and aspires to replicate the brain’s intellectual abilities (1). In general, a remarkable and sought-after ability of the brain is its *adaptive* intelligence, which enables the brain to regulate its functions “on the fly” to effectively cope with ever-changing environments, situations, goals, and rewards (2–4). A specific instance of this adaptive intelligence is context-awareness, which is known to be the hallmark of cognition or high-level intelligence (1, 5). Context-awareness is the ability of an intelligent agent to adapt its response to a certain situation or alter its decision in a scenario depending on underlying circumstances labeled “context” (6). It allows the brain to make complex, high-level decisions while considering multiple factors, to understand situations, react to new stimuli, and make predictions from small datasets (7, 8). Another adaptive cognitive function found in the brain is cross frequency coupling (CFC), which enables cognitive control of low-level neural signals by high-level knowledge (20). One flavor of CFC is phase-amplitude CFC, where the phase of a global, low-frequency neural oscillation controls or adapts the amplitude of local, high-frequency neural oscillations, where neural oscillations are rhythmic activities in the brain that occur at all levels of neural organization (9). CFC enables the brain to dynamically restructure its internal network, integrate various functional systems, and control internal signaling (10–14). A third adaptive ability called feature binding, enables the brain to combine different features of a perceived object into a coherent whole and integrate multimodal information to build a coherent representation of the external world (15). It is believed that in the brain, the phase of a global, low-frequency neural oscillation binds the features by controlling or adapting the frequency of local, high-frequency neural oscillations (16–21). Feature binding is known to be important for visual cognition (22) and is related to consciousness (23). The realization of these cognitive abilities in neuromorphic computers can lead to artificial intelligence (AI) with transformative impact (24, 25).

The adaptive abilities of human intelligence described earlier originate in the adaptive ability of biological neurons along with the plasticity of biological synapses (26) mediated by collective dynamics of neural circuits. An adaptive neuron is one in which the neuron’s properties, including its state, its transfer function, and its state space, can be altered or modulated “on-the-fly” (27, 28). A biological neuron is adaptive via neuromodulation, whereby a modulatory input to the neuron acts as a control signal and alters the neuronal properties to adapt its functioning to different situations (29, 30). Through neuromodulation, the brain can alter the amplitude and frequency of neural oscillations. This adaptive ability of biological neurons, specifically neuromodulation of neural oscillations, is critical for many additional cognitive processes, including information transfer, decision-making (31), memory (32), object representation (33–35), visual perception (36, 37), and attention (33, 38).

In addition to neuroscience, the importance of adaptive neurons is also well-recognized in software-based artificial neural networks (ANNs) and machine learning (27). According to the universal approximation theorem (39), any continuous function can be represented by an ANN using conventional, nonadaptive neurons (labeled nonadaptive ANN in this work) to a degree of approximation while using one or more hidden layers and a finite number of weights. The approximation can be improved by increasing the number of hidden layers and the network size. In contrast, the Kolmogorov–Arnold representation theorem (40) implies that in an ANN built using adaptive neurons (labeled adaptive ANN in this work) where the nonlinearity of the transfer function of every adaptive neuron can be selected, any continuous function can be represented exactly with an ANN using only one hidden layer. It is noteworthy that using adaptive neurons in an ANN allows a continuous function to be represented exactly (not just approximately) while using only one hidden layer. This implies that, in general, the expressive power of adaptive neurons in representing continuous functions supersedes that of nonadaptive neurons. In software-based ANNs, neurons with adaptive states and adaptive transfer functions have been implemented and shown to achieve superior classification performance with smaller ANN architectures than conventional nonadaptive neurons (27, 28, 41–43).

Despite the significant advantages of adaptive neurons, state-of-the-art hardware-based neurons are typically nonadaptive, i.e. most spiking neurons and all oscillatory neurons developed in hardware have a fixed state and transfer function (44–48). While there have been a few reports of neuromodulation in hardware-based spiking neurons (49–53), neuromodulation in hardware-based oscillatory neurons is yet to be achieved. Among the three cognitive abilities mentioned earlier, context-awareness and CFC have not yet been realized in hardware-based neurons while feature binding has recently been realized in just a few hardware-based neurons (54).

Here, we design and simulate a previously unachieved, hardware-based adaptive neuron that incorporates both neuromodulation and neural oscillations. We utilize this neuron to realize the three cognitive abilities mentioned earlier, namely, context-awareness, CFC, and feature binding. Additionally, the adaptive neuron is used to construct an adaptive ANN and perform context-aware diagnosis of breast cancer. Our simulations show that this adaptive ANN achieves diagnosis with higher accuracy while learning faster and using a more compact and energy-efficient architecture than nonadaptive ANNs. This work further describes how hardware-based adaptive neurons are a fundamental improvement over conventional neurons and can mitigate several important challenges facing contemporary ANNs by enabling faster learning, compact architectures, realization of broader AI, energy-efficiency, and fault-tolerance.

This adaptive neuron, named Tunable-SKyrmion-based Oscillating NEuron (T-SKONE), is designed using an artificial skyrmion lattice with five skyrmions hosted in a bilayer of insulating thulium iron garnet (TmIG) and platinum (Pt). The skyrmion lattice acts as a “*smart*” material that *adapts its structure and properties in response to an external modulatory or control input*. Simulations performed with MuMax3 (55) micromagnetic modeling show that when excited by an oscillating magnetic field, the neuron produces spin waves originating from skyrmion oscillations. The spin waves demonstrate a multifrequency spectrum that results from skyrmion–skyrmion coupling (56, 57). This neuronal spectrum consists of four distinct resonant modes, identified as counterclockwise gyration, breathing, and the hybridizations of both. The control input consists of an external current that re-arranges the locations of skyrmions in the lattice. This reconfiguration of the skyrmion lattice alters both the amplitude and frequency of the neuronal output, thereby altering the neuron’s state, transfer function, and state space, emulating neuromodulation.

T-SKONE is designed using an electrically reconfigurable lattice of skyrmions since spintronic nanodevices are highly attractive for neuromorphic computing due to their small footprint, high endurance, and low power consumption (58, 59). Moreover, skyrmions are particularly beneficial due to their small size and their ability to be created, manipulated, detected, and erased by current or field (60). However, the concept of leveraging smart materials to mimic neuromodulation is general and adaptive neurons could be realized with a variety of material systems including memristive, ferroelectric, and magnetic domain wall materials (61–66).

T-SKONE is used to realize context-awareness in hardware, which is demonstrated with micromagnetic simulations. Here, the contextual information is fed into T-SKONE via the neuron’s control input, such that changes in context alter the neuron’s state, transfer function, and its response. To demonstrate the potential of this context-aware ability of T-SKONE, NN simulations are conducted to perform context-aware diagnosis of breast cancer. In these simulations, an adaptive ANN is constructed using neurons modeled after T-SKONE. Feature characteristics of breast mass biopsies are fed into the ANN as direct or conventional input and contextual patient medical data are fed into the ANN as control input. These simulations are benchmarked against nonadaptive ANNs that diagnose cancer by fusing the biopsy feature data and contextual medical data without using adaptive neurons in a method known as multimodal, multivariate data fusion (67, 68). The adaptive ANN is shown to outperform the nonadaptive ANN as it learns faster and diagnoses cancer with higher final accuracy, while requiring a smaller architecture and less energy. Breast cancer was chosen as the task as it is one of the most common cancers in women, affecting 25% of all women with cancer worldwide (69) and early and accurate diagnosis of the disease enhances the survival rate (69).

The second cognitive ability shown by T-SKONE, CFC, is demonstrated by feeding a low-frequency wave into the control input of the neuron such that the phase of this wave modulates the amplitude of the high-frequency neuronal output. Taking inspiration from the brain where CFC is used to dynamically restructure the internal NNs, we present the design of a structurally flexible, hardware-based ANN constructed from T-SKONEs. In this flexible ANN, CFC is utilized to dynamically switch the adaptive neurons “on” or “off,” so as to reconfigure the network topology either on a preselected basis or at run-time. Flexible networks provide a general problem-solving tool that can be reconfigured for optimal solution of one task or across several tasks (70).

The third cognitive ability shown by T-SKONE, namely, feature binding, is demonstrated by feeding a low-frequency wave into the control input of the adaptive neuron such that the phase of this wave modulates the frequency of the high-frequency neuronal output. A network of T-SKONEs receives visual information about two objects, a “magenta circle” and a “blue cross.” Two different features (shape and color) of the same object are encoded by the neurons at the same frequency such that they can be correctly bound together via synchronization to construct a coherent percept of the original object.

Therefore, hardware-based adaptive neurons can realize previously unachieved cognitive capabilities with far-reaching applications. Context-aware computation demonstrated here can have sweeping impact on human–machine collaboration, personalized healthcare, advanced manufacturing, and education (25, 71–74). Similarly, CFC-based flexible ANNs presented here can achieve energy-efficient and compact AI, which upon integration with 5G and IoT technologies would lead to smart agents on the edge impacting numerous facets of everyday life (75). Moreover, feature binding enables multimodal information fusion, which can transform autonomous vehicles, neuro-prosthesis, wearable health technology, agriculture, and climate control (76–79).

More generally, hardware-based adaptive neurons can mitigate several critical challenges facing contemporary ANNs. Modern ANNs demand huge volume of training data (80, 81), unsustainably large energy, and chip area when implemented on CMOS circuits (82) and are highly specific in their applications (24, 83). In contrast, adaptive neurons like T-SKONE can mitigate these challenges by enabling ANNs that learn faster, have compact architectures, are energy-efficient, are fault-tolerant, and realize broader AI.

## Results

### Design of hardware-based adaptive oscillatory neuron

**Fig. 1.**
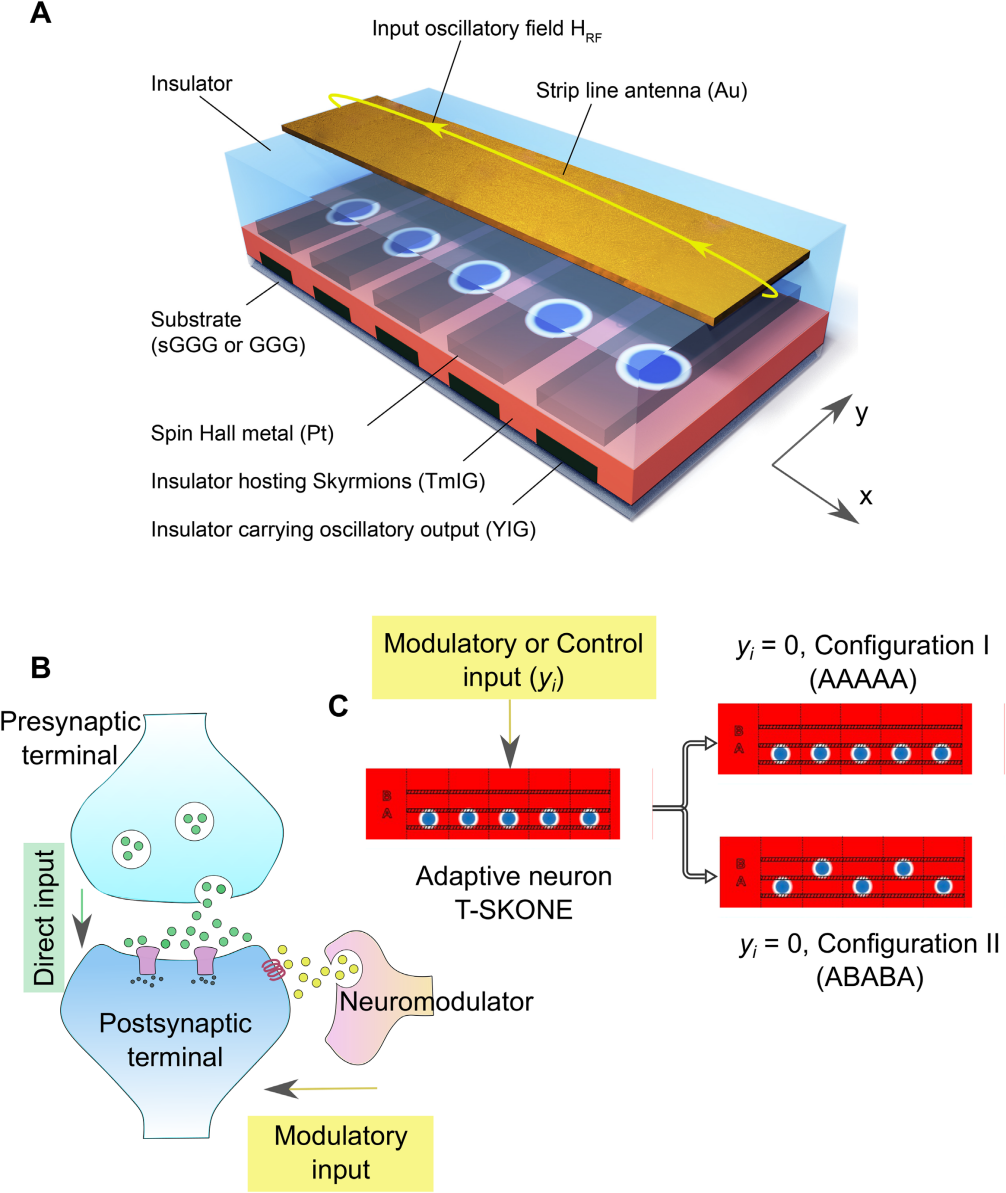
Schematic of the adaptive oscillatory neuron. (A) Structure of the neuron. TmIG (salmon): insulating magnet hosting the skyrmion lattice. ${H_{RF}}$: excitatory magnetic field input carried by the strip line antenna. ${J_1}\hbox{--}{J_5}$: modulatory current inputs carried by the spin Hall metal (Pt). YIG: spin wave output readout. (B) Schematic for neuromodulation in the brain. Direct input is carried from the presynaptic terminal to the postsynaptic terminal via neurotransmitters (green circles) and modulatory input (yellow circles) is carried from the neuromodulator to the postsynaptic terminal. (C) Schematic for neuromodulation in the skyrmion lattice. The control input (*y_i_*) reconfigures the skyrmion lattice, with skyrmions (blue circles) arranged in Configuration I (AAAAA, top) for *y_i_* = 0, and II (ABABA, bottom) for *y_i_* = 1. The shadowed regions have reduced PMA, designed to softly pin the skyrmions.

A schematic of T-SKONE is shown in Fig. 1A. The skyrmion lattice comprises five nanotracks, (with a nanotrack along *y*), each hosting a single skyrmion (blue). The five skyrmions are labeled *Sk1–Sk5* along increasing *x*, as shown in Fig. 1A. The neuron is designed to mimic neuromodulation (see Fig. 1B). To precisely arrange the skyrmions in the lattice and prevent skyrmion dislocation upon application of an oscillatory magnetic field, reduced perpendicular magnetic anisotropy (PMA) regions are used as skyrmion pinning sites (shadowed regions in Fig. 1C). Such reduced PMA regions can be created using ion implantation (84). Since the skyrmion boundary with in-plane magnetization tends to be located in the low-PMA regions to reduce the overall magnetostatic energy (85), the skyrmions can be softly pinned into lattice sites A or B (Fig. 1C). The neuron receives two types of inputs: (i) direct or driving input in the form of an excitatory magnetic field input (*H*_RF_) along *x* applied to all skyrmions through a gold strip line antenna, which induces localized skyrmion oscillations and produces spin waves (86, 87), and (ii) control or modulatory input in the form of charge currents (${J_1} \hbox{--} {J_5}$) along *y* applied to individual skyrmions through the spin Hall metal Pt overlayers (purple), which independently move the skyrmions along the nanotracks and position them in site A or B. As the skyrmion lattice configuration is modified (e.g. between Configurations I and II as shown in Fig. 1C), so are the skyrmion coupling strengths and therefore their resonant frequencies and amplitudes of oscillation. Thus, the response of the neuron to the direct input can be modulated by the control input, mimicking neuromodulation. The outputs from the skyrmion nanotracks can be read from inductive antennas placed below individual nanotracks in the form of AC voltages or carried by coplanar waveguides composed of an insulating magnet (YIG) in the form of spin waves.

Recent work has reported the observation of above and near room-temperature skyrmions in a bilayer heterostructure composed of a magnetic insulator, thulium iron garnet (TmIG, Tm_3_Fe_5_O_12_) in contact with a spin Hall metal (Pt) (88). There have also been experimental observations of domain wall motion in TmIG/Pt bilayers driven by spin orbit torque (89, 90). The TmIG/Pt system provides innate electrical isolation to the individual nanotracks, while at the same time allowing the skyrmions to couple magnetically. However, since the skyrmion phase in TmIG/Pt typically exists at and above 360 K, more work is required for the design of room-temperature stable insulating skyrmion materials.

### Simulation of the adaptive neuronal function

**Fig. 2.**
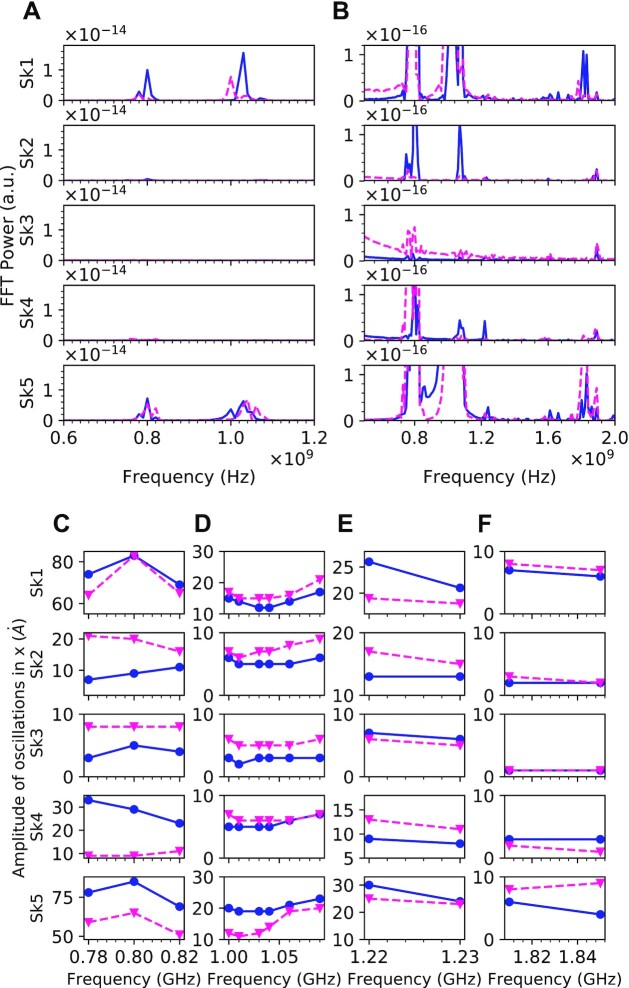
Coupled dynamics of T-SKONE. (A) Frequency modulation: FFT power density spectra of the neuron for Configuration I (solid blue line) and Configuration II (dashed magenta line), for all five skyrmions (*Sk1*–*Sk5*). (B) Zoomed-in plot of (A). (C) to (F) Amplitude modulation: amplitudes of oscillation for all five skyrmions shown at a range of frequencies for Configuration I (blue circles) and Configuration II (magenta triangles).

The oscillatory dynamics of T-SKONE was simulated for two lattice configurations, Configuration I (AAAAA) and II (ABABA), using the MuMax3 solver (55), as described in Supplementary Material S1. The fast Fourier transform (FFT) power density spectra of the skyrmion core trajectories was calculated (see Supplementary Material S1) and is shown in Fig. 2A and B, with Configuration I in solid blue and Configuration II in dashed magenta. As the configuration of the neuron changes, the peaks of the spectrum that signify the resonant frequencies also shift, which demonstrates that the neuron is capable of frequency modulation. Fig. 2A shows two resonant peaks for both configurations at around 0.80 and 1.04 GHz corresponding to Mode 1 and Mode 2, respectively. The FFT spectra when zoomed-in and extended up to 2.0 GHz is shown in Fig. 2B, where two more resonant peaks around 1.22 GHz (Mode 3) and around 1.85 GHz (Mode 4) can be seen. In total, there are 2^5^ neuronal configurations available in this design and more work is required to determine the configurations with notably distinct dynamics. T-SKONE is estimated to utilize ultralow power of <0.1 pJ/oscillation at 1 GHz and is ultracompact at 1200 nm length compared to a CMOS neuron (265 pJ/oscillation, >30 μm) and a spin-torque oscillator (3 pJ/oscillation, 300 nm) (91), while being significantly more computationally powerful. The power utilized by T-SKONE is estimated based on the driving current in the strip line antenna required to produce an external AC magnetic field of $10\;Oe$ and a DC magnetic field of $240\;Oe$, with the antenna assumed to be 300 nm above the neuron.

To demonstrate amplitude modulation, the neuron was excited by a sinusoidal magnetic field as described in Supplementary Material S1. Fig. 2C to F shows the amplitudes of oscillations of the skyrmion cores along *x* at driving frequency ranges centered on (C) Mode 1, (D) Mode 2, (E) Mode 3, and (F) Mode 4. There are multiple instances of a clear difference in the amplitudes of oscillations at a given frequency for Configurations I (solid blue) and II (dashed magenta), which demonstrates amplitude modulation. For instance, at 0.80 GHz (Mode 1), *Sk3* has a larger amplitude of oscillation for Configuration II than I, while *Sk5* shows a larger amplitude for Configuration I than II, both of which are consistent with the FFT power spectrum. It is worth noting that the small driving field ($10\;Oe$) required to induce large oscillation amplitudes (up to 8 nm) is highly promising for ultralow power operation of the neuron. Amplitude modulation along *y* is shown in Supplementary Material S2 and Fig. S1.

**Fig. 3.**
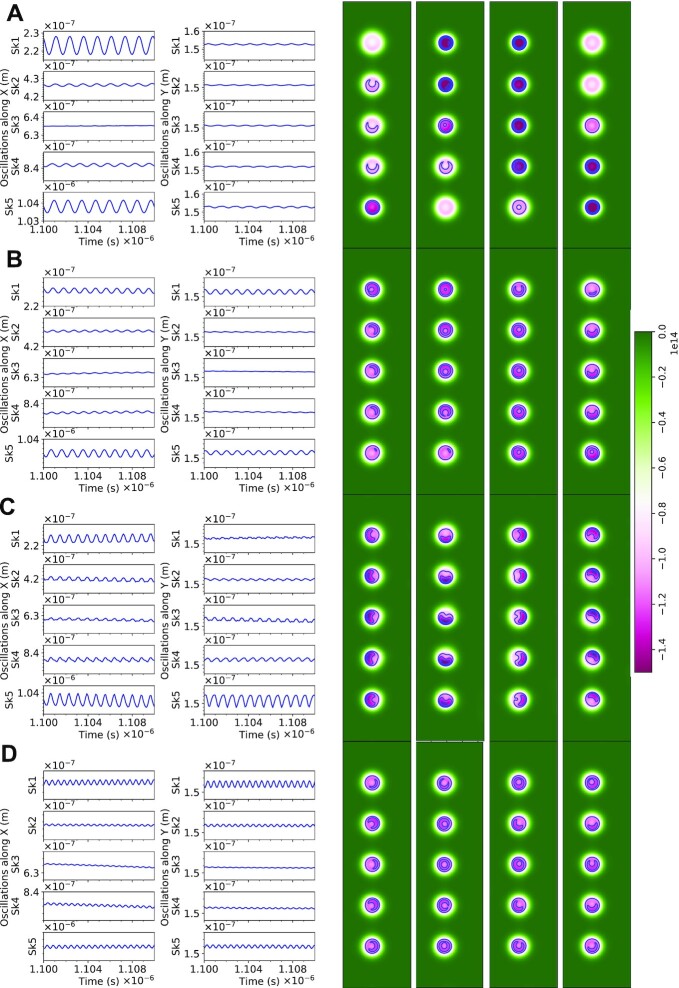
Resonant modes of T-SKONE in Configuration I when excited by a sinusoidal driving field of frequency (A) 0.80 GHz (Mode 1), (B) 1.04 GHz (Mode 2), (C) 1.22 GHz (Mode 3), and (D) 1.85 GHz (Mode 4). The two left most columns plot the output oscillations along *x* and *y*, respectively. The four columns on the right plot the topological charge densities at times $t\; = \;0$, $T/4$, $T/2$, and $3T/4$, where $T$ is the respective time period of oscillation. The color maps for topological charge density ranges from $ - 1.5 \times {10^{14}}\,{m^{ - 2}}$ (magenta) to zero (green).

We now discuss the four resonant modes identified above in more detail. Fig. 3 shows the oscillatory response of the neuron while in Configuration I, upon excitation with a sinusoidal magnetic field. The first and second columns of Fig. 3A to D plot the oscillations for every skyrmion core along *x* and *y*, respectively, for resonant modes (1–4). The skyrmion cores oscillate sinusoidally with frequencies locked to the driving input and with a wide range of amplitudes. To reveal the physical origin of these modes, topological charge density maps of the T-SKONE are plotted at times $t\; = \;0$, $T/4$, $T/2$, and $3T/4$, where $T$ is the respective time period of oscillation. These maps are shown in the four columns on the right of Fig. 3 and demonstrate that every resonant mode originates from a distinct type of skyrmion dynamics. In general, the oscillation amplitudes are larger for the outer skyrmions (*Sk1* and *Sk5*) than amplitudes for inner skyrmions (*Sk2*–*Sk4*). This is likely because the dominant form of interaction is the skyrmion–skyrmion repulsion, which suppresses the oscillations of the inner skyrmions.

Fig. 3A shows the skyrmion oscillation for Configuration I at Mode 1 (0.80 GHz). The topological charge density plots demonstrate that this is a breathing mode in which the skyrmion cores periodically expand and contract in size. At this frequency, the skyrmions are out-of-phase with their neighbors, which is favored by the skyrmion–skyrmion repulsion. Fig. 3B plots the output for Configuration I at Mode 2 (1.04 GHz). From the topological charge density maps, a mixture of the two oscillations instead of complete cycles of breathing mode or gyration mode are observed over the time period of oscillation, which indicates that this mode is a hybridization between these two pure modes. The amplitudes for the inner skyrmions (*Sk2*–*Sk4*) are comparable, and their oscillations while not perfectly phase-locked are similar in their phase ordering. This is expected due to the hybridized nature of Mode 2.

Skyrmion oscillations at Mode 3 (1.22 GHz) are shown in Fig. 3C. Topological charge density plots reveal the origin of this mode to be a CCW gyration mode with all the skyrmions synchronized at 1.22 GHz. Finally, Fig. 3D demonstrates oscillatory dynamics at Mode 4 (1.85 GHz) where oscillations for all skyrmions are generally small (<1 nm). Topological charge density maps reveal this to be a highly complex mode originating from the hybridization of breathing, counterclockwise, and clockwise gyration.

With few exceptions, the largest oscillatory amplitudes were seen for Modes 1 and 3 and the smallest were seen for Mode 4. This is likely because Modes 1 and 3 are pure modes, whereas Modes 2 and 4 are hybridizations of the pure modes. The frequency range corresponding to Mode 2 lies between that of the pure Modes 1 and 3, since Mode 2 is a hybridization of Modes 1 and 3. The discovery of hybridized modes in skyrmion lattices brings a particularly rich and coupled dynamics to the oscillatory neuron, enhancing its utility for neuromorphic applications. When the neuron in Configuration II was excited with the same frequencies as above, it also demonstrated four modes with the same physical origin, but with different oscillation amplitudes (see Supplementary Material S3 and Fig. S2). Additionally, amplitude modulation and frequency modulation were demonstrated using the metallic CoFeB/heavy metal bilayer system (see Supplementary Material S4, Figs. S3 and S4).

### Design of an ANN using T-SKONE

**Fig. 4.**
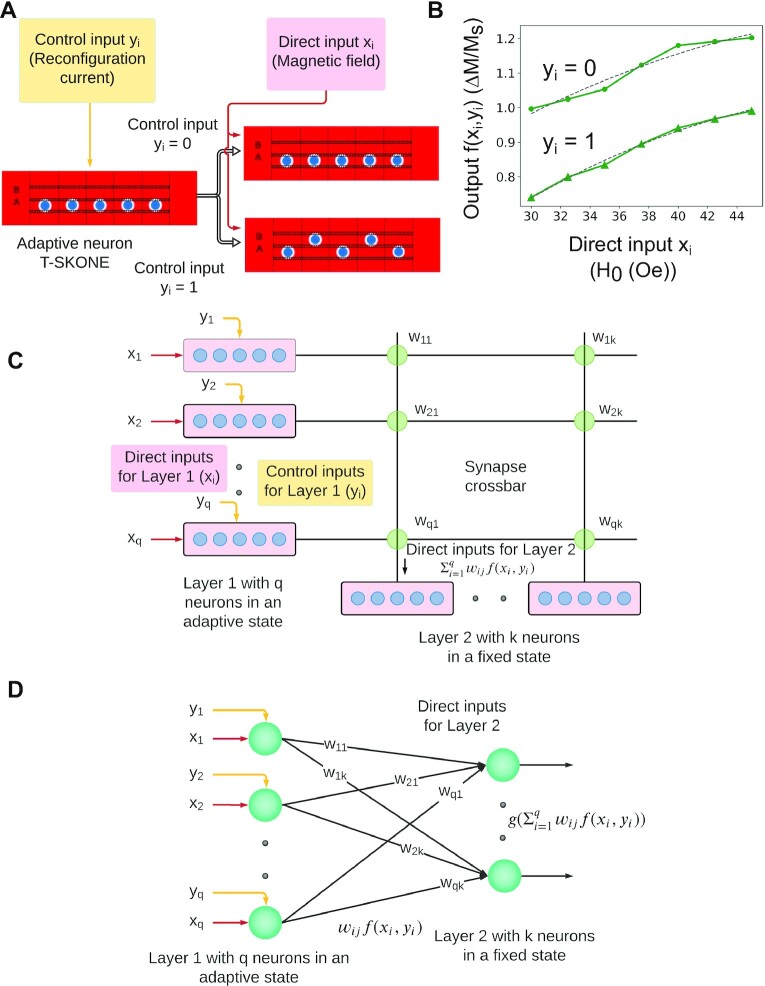
Mapping of skyrmion arrays (T-SKONEs) into an adaptive ANN. (A) Schematic for adaptive behavior in a single T-SKONE. The neuron receives a control input *y_i_* that reconfigures the neuronal state. (B) Transfer function *f*(*x_i_*, *y_i_*) for the adaptive neuron T-SKONE, which is modified by the control input *y_i_*. The transfer function is calculated using micromagnetic simulations (green) and converted into an analytical model (gray) that is used for NN simulations. (C) Diagram for a hardware ANN using T-SKONEs. In layer 1, T-SKONEs receive two inputs, direct (*x_i_*) and control (*y_i_*) with the latter modulating the neuronal state. The outputs from layer 1 are fed into layer 2 via spintronic synapses and T-SKONEs in layer 2 are kept in a fixed state. (D) The equivalent network model for the ANN shown in (C).

The input–output characteristics of T-SKONE obtained using micromagnetic simulations are now mapped into a model adaptive neuron and an ANN is constructed using this neuron, as shown in Fig. 4. Since T-SKONE has many possible configurations and its output can be selected from any of the five skyrmion nanotracks, the exact neuronal outputs and the network architecture will depend on the specific task at hand. However, this section presents a general method used to construct an ANN using the adaptive neurons.

Here, we consider a fully connected, feed-forward ANN with two layers. Neurons in the first layer are in an adaptive state and receive two inputs, a conventional or direct input (*x_i_*) and a control or modulatory input (*y_i_*). The direct input (*x_i_*) is fed to T-SKONE (*T_i_*) encoded in the amplitude of a sinusoidal magnetic field (*H*_RF_) with a frequency of 0.80 GHz. This field excites the breathing mode of the skyrmion lattice in T-SKONE. The control input (*y_i_*) is fed to T-SKONE encoded in modulatory currents (${J_1} \hbox{--} {J_5}$) that reconfigure T-SKONE and alter its state, as shown in Fig. 4A. Control input *y_i_ *= 0 and 1 corresponds to Configuration I and II, respectively.

Here, the output of T-SKONE is designed to be detected by an inductive antenna placed below the nanotrack for skyrmion 4 (fourth skyrmion along *x* in Fig. 1A). This induced AC voltage is proportional to the average change in magnetization in the nanotrack $\Delta M$ (${V_{peak}} \approx {\mu _0}\Delta MA\varpi $) (92), where ${\mu _0}$ is the vacuum permeability, *A* is area of the antenna, and $\varpi $ is the oscillation frequency. Therefore, $\Delta M/{M_s}$ is used as the effective T-SKONE output in this task, where ${M_s}$ is the saturation magnetization. This effective output was calculated using micromagnetic simulations by averaging the change in magnetization over four distinct regions of the nanotrack, i.e. the skyrmion core, the skyrmion boundary, the region between the core and boundary, and the region of the nanotrack outside the skyrmion. The neuronal output ($\Delta M/{M_s}$) plotted in Fig. 4B in green is notably different for different control inputs (*y_i_* = 0,1). Therefore, the control input modulates the state and transfer function of the adaptive neuron. The neuron’s input–output characteristics were fitted to a polynomial transfer function (plotted in Fig. 4B in gray) to obtain a model transfer function. The transfer function *f*(*x_i_*, *y_i_*) for *i*th neuron is given by

$$
\begin{equation*}
f\;\left( {{x_i},{y_i}} \right) = {\rm{\;}} \bigg \{ \begin{array}{@{}*{1}{c}@{}} { - 0.00038{x_i}^2 + {\rm{\;}}0.04413{{\rm{x}}_i} + 0.00300,{\rm{\;}}for{\rm{\;\;}}{y_i} = {\rm{\;}}0}\\ { - 0.00049{x_i}^2 + {\rm{\;}}0.05347{{\rm{x}}_i} - 0.42914,{\rm{\;}}for{\rm{\;\;}}{y_i} = {\rm{\;}}1} \end{array}.
\end{equation*}
$$

The outputs of the neurons in the first layer $f( {{x_i},{y_i}} )$ are sent through synaptic weights ${w_{ij}}$, added and sent as direct input to neurons in the second layer, as shown in Fig. 4C.

In this general example, the neurons in the second layer are T-SKONEs (skyrmion arrays) kept in a fixed state, namely, Configuration I. The input received by the *j*th neuron of the second layer is ${u_j} = \mathop \sum \limits_{i = 1}^q {w_{ij}}f( {{x_i},{y_i}} )\;$. The transfer function *g*(*u_j_*) of the *j*th neuron of the second layer is obtained by mapping the input–output characteristics of T-SKONE in Configuration I to a polynomial function and is given by

$$
\begin{equation*}
g\;\left( {{u_j}} \right) = {\rm{\;}} - 0.00347{u_j}^3 + \;0.34476{u_j}.
\end{equation*}
$$

We note that *g*(*u_j_*) is not equal to $f( {{x_i},\;{y_i} = \;0} )$, even though in both cases T-SKONE is in Configuration I. This is because the range of direct inputs for layer 1 (*x_i_*) and layer 2 (*u_j_*) are different. In the first layer, *x_i_* is restricted to values between 30 and 45 Oe to prevent any overlap between the output values seen for the two configurations, while in the second layer *u_i_* can take any value between 0 and 45 Oe. Therefore, *g*(*u_j_*) and $f( {{x_i},\;{y_i} = \;0} )$ are fitted to functions with different ranges. The final output of the *j*th neuron of the second layer is $g( {\mathop \sum \limits_{i\; = \;1}^q {w_{ij}}f( {{x_i},{y_i}} )} )$.

In a hardware implementation of this ANN, using $\Delta M\;\sim\;{M_s}$(saturation magnetization) gives an estimated AC output voltage for T-SKONE in the range of 0.01 mV. This AC output can be amplified and fed to a crossbar of domain wall-based synapses that implement the stochastic gradient descent learning algorithm (93) and apply weights *w_ij_*. The output AC voltages from this synaptic layer can be fed as direct inputs to the second neural layer in the ANN. The need for amplification, which hampers the energy efficiency of this network, can be mitigated by using skyrmion nanotracks with larger ${M_s}$ or designing spin wave-based synapses that directly connect subsequent T-SKONE layers without using inductive antennas.

### Demonstration of context-aware diagnosis of breast cancer using T-SKONE

Context-awareness is a key ability of the brain, where context refers to information that characterizes the situation of an entity, e.g. place, time, emotional state, etc (94). In this work, context-aware tasks are defined as tasks where classification or prediction is performed using (i) direct information, which is defined as the data that are being classified; and (ii) contextual information, which is defined as background data that are related to the direct information but is not itself being classified. As an example, here we implement context-aware diagnosis of breast cancer where biopsy image features of breast mass are classified to predict whether the biopsy results are malignant or benign and patient’s medical data provide important background information to help make the diagnosis. Therefore, the biopsy image features that are being classified are direct information, and the patient’s medical data are contextual information. Context-awareness includes a large class of applications encompassing brain–machine interfaces, biomedicine, IoT, robotics, etc.

Due to the absence of a well-known, multimodal cancer dataset containing information about both biopsy images and patient’s medical data, we constructed such a dataset by appending the Breast Cancer Wisconsin (Diagnostic) dataset (comprising biopsy image features) (95) with information regarding patient’s medical data (comprising attributes *α_i_*). The four selected attributes selected were: (i) patient is female, (ii) patient is older than 50 y, (iii) patient has a body mass index in the obese range, and (iv) patient has a large intake of alcohol. The modified dataset was constructed to represent statistical correlations seen in reality, details about which are described in Supplementary Material S5 and Table S1. Breast cancer was selected as the task since it can be implemented as a context-aware problem unlike more commonly used benchmarking approaches like the MNIST image recognition task. In addition, breast cancer is selected since it is the most commonly diagnosed cancer in women, affecting 2.1 million women each year and is the leading cause of female cancer deaths. Early and accurate detection can enhance survival rates among breast cancer patients (96).

State-of-the-art in machine learning for context-aware cancer diagnostics uses multimodal data fusion (67, 68, 97), where both biopsy image features and patient’s medical data are fed as direct input to nonadaptive neurons. To implement this method, we constructed an ANN that uses T-SKONEs kept in a fixed state (Configuration I). The ANN comprised a fully connected, feed-forward network with no hidden layers. The input layer comprised a total of 34 neurons, out of which 30 neurons were fed the biopsy data and the remaining 4 were fed the medical data, both as as direct input (*x_i_*) (see Fig. S5 in Supplementary Material). Feature characteristics of biopsies were encoded in the amplitude of a sinusoidal magnetic field (*H*_RF_) with a frequency of 0.80 GHz and a range of values between 30 and 45 Oe. The medical data being binary in nature, were encoded in the direct input such that *α_i_* = “F” or “T” corresponded to *x_i_* = 0 or 1, respectively. Thus, layer 1 of the network consisted of a total of 34 neurons, with 30 neurons that processed the biopsy feature data and had a state space of [0.996, 1.202] $\Delta M/{M_s}$ and 4 neurons that processed medical data and had a state space of [0.996, 1.202] $\Delta M/{M_s}$. The *i*th neuron of the input layer had the transfer function $f^{\prime}\;( {{x_i}} ) = \; - 0.00038{x^2} + \;0.04413{\rm{x}} + 0.00300\; = \;f( {{x_i},{\rm{\;}}{y_i} = \;0} )$.

**Fig. 5.**
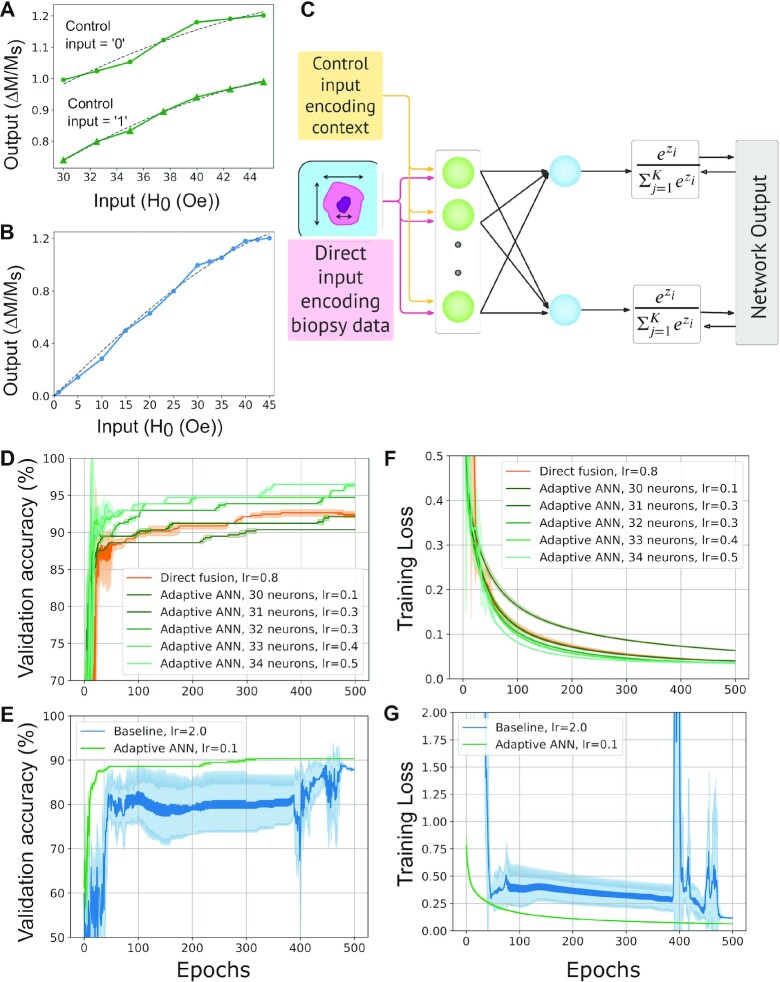
Context-aware diagnosis of breast cancer using T-SKONE. (A) Transfer function for T-SKONE used in Layer 1. The control input (*y_i_*) modulates the configuration of the skyrmion array and alters the neuron’s transfer function. (B) Transfer function for T-SKONE in fixed state used in Layer 2. (C) Diagram of adaptive ANN used for diagnosis, with neurons in Layer 1 (blue circles) receiving biopsy image data (direct input) and patient’s medical data (control input). (D and E) Validation accuracy and (F and G) loss of information for adaptive ANN (green), direct data fusion (orange), and baseline ANN (blue). The adaptive ANN consistently learns faster from smaller amounts of data and achieves higher accuracy while using a smaller network. (A, C, E, and G) are created in Lucidchart [134].

The output from the input neurons was fed to a series of software synapses that were treated as ideal double-precision weights *w_ij_*. The synapses were trained by applying the Adam optimization algorithm (98), a form of adaptive momentum stochastic gradient descent. The synaptic output was fed as direct input to neurons in the output layer, which were also modeled after T-SKONEs kept in a fixed state (Configuration I). The input into a *j*th neuron was ${u_j} = \mathop \sum \limits_{i = 1}^q {w_{ij}}{f_i}( {{x_i},{y_i}} )\;$ and the range of this input was kept between 0 and 45 Oe by scaling synaptic weights. Neurons in the output layer had a state space of [0, 1.202] $\Delta M/{M_s}$ and a transfer function $g\;( {{u_j}} ) = {\rm{\;}} - 0.00347{u_j}^3 + \;0.34476{u_j}$, which is shown in Fig. 5B. This simulation was labeled “direct data fusion” and formed the benchmark for our calculations.

In addition to the benchmark simulation described above, we carried out context-aware cancer diagnosis with an ANN built from adaptive neurons. In general, context-aware tasks can be implemented using adaptive neurons like T-SKONE by encoding the contextual information in the neuron’s control input. Changes in context, alter the control input, which, in turn, alters the neuron’s state, transfer function and therefore the neuronal response, implementing context-awareness. In this specific task, an adaptive ANN was constructed from T-SKONEs in the same manner as the general method described previously (see Fig. 4). Analogous to the direct data fusion simulation described above, the ANN here comprised a fully connected, feed-forward network with two layers. Feature characteristics of biopsies were fed to T-SKONE (*T_i_*) in the first layer as direct input (*x_i_*) encoded in the amplitude of a sinusoidal magnetic field (*H*_RF_) with a frequency of 0.80 GHz and a range of values between 30 and 45 Oe. However, the patient’s medical data was fed to T-SKONE (*T_i_*) in the first layer now as control input (*y_i_*), such that *α_i_* = “F” or “T” corresponded to *y_i_* = 0 or 1, respectively. This simulation was labeled “context-aware.”

According to the universal approximation theorem (39), increase in the size of a NN can improve the accuracy with which the network represents a function. Thus, a fair initial comparison between the adaptive (context-aware) network and the benchmark (direct data fusion) network, necessitated that the two networks be of equal size. The direct data fusion network required 34 input neurons and 68 synapses, since 30 input neurons were fed biopsy image attributes (as *x_i_*) and 4 input neurons were fed the medical data attributes (also as *x_i_*). By comparison, the context-aware network required only 30 input neurons and 60 synapses since the biopsy data were fed to 30 input neurons as direct input (*x_i_*) and the medical data were fed to these neurons as contextual input (*y_i_*). The contextual inputs (α_1_, α_2_, α_3_, and α_4_) were fed into seven, seven, eight, and eight input neurons, respectively. However, to equalize the size of the two networks, four dummy input neurons (and a corresponding eight synapses) were added to the context-aware network, where these dummy neurons received a constant input.

The state space of the *i*th T-SKONE in the first layer (*T_i_*) of the context-aware network is given by

$$
\begin{equation*}
\bigg \{ \begin{array}{@{}*{1}{c}@{}} {{\rm{\;}}\left[ {0.996,{\rm{\;}}1.202} \right]{\rm{\;}}\Delta M/{M_s},{\rm{\;}}for{\rm{\;\;}}{y_i} = {\rm{\;}}0}\\ {\left[ {0.740,{\rm{\;}}0.990} \right]{\rm{\;}}\Delta M/{M_s},{\rm{\;}}for{\rm{\;\;}}{y_i} = {\rm{\;}}1} \end{array}.
\end{equation*}
$$

As before, T-SKONE was mapped onto a model neuron by fitting the former’s input–output characteristics to a polynomial transfer function, which is plotted in Fig. 5A and is given by

$$
\begin{equation*}
f\;\left( {{x_i},{y_i}} \right) = {\rm{\;}}\bigg \{ \begin{array}{@{}*{1}{c}@{}} { - 0.00038{x_i}^2 + {\rm{\;}}0.04413{{\rm{x}}_i} + 0.00300,{\rm{\;}}for{\rm{\;\;}}{y_i} = {\rm{\;}}0}\\ { - 0.00049{x_i}^2 + {\rm{\;}}0.05347{{\rm{x}}_i} - 0.42914,{\rm{\;}}for{\rm{\;\;}}{y_i} = {\rm{\;}}1} \end{array}.
\end{equation*}
$$

Since the samples were classified as either malignant or benign, Layer 2 of both networks consisted of two neurons. Layer 2 was kept the same as for the nonadaptive ANN described in the direct data fusion simulation.

For robust benchmarking, the learning rate was individually optimized for every simulation. The NN simulation was implemented in PyTorch (99). Total 20% of the samples were separated from the training set and used for validation. The network training was applied for 800 epochs. Results from these simulations are plotted in Fig. 5D to F, with direct data fusion plotted in orange and context-aware simulation shown in light green. The accuracy and loss results were averaged across five randomized weight initializations. The shaded regions of Fig. 5D to G show the standard deviation of the relevant metric at a particular epoch.

As can be seen from Fig. 5D, the final accuracy of diagnosis attained by direct data fusion is 92%, while that obtained by the context-aware network is 97%. Thus, the context-aware network achieves much higher accuracy of cancer diagnosis than a nonadaptive network of comparable size. Subsequently, we reduced the size of the context-aware network by removing the dummy neurons one by one with the simulation results plotted in Fig. 5D. As expected, the performance of the context-aware network degrades as it is made smaller, at least in part because it has fewer synapses or weights with which to accurately represent the classification function. Even with this smaller size, the context-aware network continues to outperform the nonadaptive network for 33 (and 32) neurons in Layer 1 along with 66 (and 64) synapses as compared to the nonadaptive network that has 34 neurons and 68 synapses. Thus, the context-aware network is able to outperform the nonadaptive network using smaller circuit size and lesser energy.

It is noteworthy that the addition of dummy neurons that receive constant inputs does not add any computational power to the context-aware network. It only serves to equalize the number of synapses in both networks, thereby equalizing their memory ability. This allows us to isolate and compare only the computational power of the two networks. Thus, our results show that the computational power of an adaptive neuron supersedes that of a nonadaptive neuron.

An additional benchmarking simulation was performed where breast cancer was diagnosed using only the baseline data of biopsy image features. The ANN was constructed exactly as the direct data fusion network described earlier, except that Layer 1 now only had 30 neurons and only took in biopsy data encoded in the direct input (*x_i_*) (see Fig. S5 in Supplementary Material). We label this simulation “baseline” and plot its results in Fig. 5E and G in blue.

A consistent feature of the ANN built from adaptive neurons is its ability to learn faster than ANNs built from nonadaptive neurons, which persists even with changes in network topology. This can be seen from the smaller optimized learning rate (*lr*) for the adaptive ANNs (0.1 ≤ *l*r ≤ 0.5) than for the nonadaptive ANNs used in direct data fusion (*lr = *0.8) and baseline (*lr *= 2.0) simulations. Additionally, the validation accuracy in the initial epochs is higher (see Fig. 5D and E) and training loss is lower (see Fig. 5F and G) for adaptive ANNs (shown in green) than for the nonadaptive ANNs, signifying faster learning by the adaptive neurons.

### Fundamental benefits of T-SKONE-based context-aware ANNs

Hardware-based adaptive neurons like T-SKONE are a fundamental advancement over nonadaptive neurons and can mitigate several challenges facing ANNs that are built using nonadaptive neurons.

#### Faster learning

A severe challenge facing conventional ANNs is their requirement of huge volumes of training data (80, 81). Our results show that an ANN with adaptive neurons consistently learns faster than nonadaptive ANNs in context-aware tasks. Moreover, in software-based ANNs, adaptive neurons have been verified to learn faster than nonadaptive neurons in a wide variety of tasks using various numerical approaches (27, 28, 41–43).

#### Energy-efficiency and compact architecture

Another significant challenge is that software-based ANNs implemented with conventional CMOS circuits use unsustainably large energy and area (82). In contrast, T-SKONE uses ultralow power and is ultracompact. Additionally, it is well known that neurons with adaptive transfer functions can express a desired function using more compact architectures than nonadaptive neurons (40). Therefore, T-SKONE-based ANNs will use less energy and chip area than software-based ANNs and many hardware-based nonadaptive ANNs.

#### Realizing broader AI

Conventional ANNs are adept at highly specific tasks and are not proficient outside that domain (24). For instance, conventional ANNs cannot effectively deal with changing environments, contexts, goals, or rewards their applications (24, 83). This “narrow” intelligence is a core challenge facing modern ANNs (24). In this work, contextual information controls the functioning of adaptive neurons enabling the ANN to deal with dynamically varying context more effectively than nonadaptive ANNs. Similarly, environmental signals, goals, and rewards can also be used to control the functioning of T-SKONE, to realize ANNs adept at performing dynamic tasks. An example of this is demonstrated for a human–machine interaction task (see Supplementary Material S6 and Fig. S6). Here, the neuron receives a direct input representing a human spoken command and a control input representing environmental safety, specifically whether a box is safe to open. Upon receiving a “go” command from the human being, the neuron can adaptively decide whether or not to open the box, depending on whether the box is safe or not. These results present a roadmap for realizing broader AI using T-SKONE-based ANNs.

Additionally, a recent breakthrough in the development of broad AI involves Neuro-symbolic AI. A combination of deep NNs and symbolic AI, neuro-symbolic AI creates symbolic models that capture the underlying relationships between factors and forces, are augmented with contextual information, and can predict situations that are yet to be encountered (100, 101). As T-SKONE-based ANNs are fundamentally superior at processing contextual information than nonadaptive ANNs, the combination of adaptive ANNs with symbolic AI could be highly attractive for realizing broader AI. Furthermore, an important concept in the search for broad AI is top–down knowledge, where “understanding” is encapsulated in feedback signals from high-level to low-level networks (102). The control input of T-SKONE is innately well-suited to carrying this top-down knowledge enabling high-level networks to modulate the functioning of low-level networks.

#### Fault-tolerance

An additional benefit of adaptive neurons is that at any time, different neurons in the ANN are in different states and use different transfer functions. Thus, the errors produced by these neurons are less correlated than in nonadaptive ANNs, which leads to greater fault-tolerance (103).

The performance of the context-aware network can be further optimized by tuning the input sequence of contextual attributes as shown in Supplementary Material S7 and Fig. S7 and a simple extension of the encoding scheme to include negative valued inputs. It is expected that this optimization can enable the context-aware network to compete with state-of-the-art machine learning networks (see Supplementary Material S8 and Fig. S8). The context-aware network is also benchmarked on the well-known MNIST task to further demonstrate the accuracy of its performance (see Supplementary Material S12 and Fig. S12).

### Demonstration of CFC and structurally flexible ANNs using T-SKONE

**Fig. 6.**
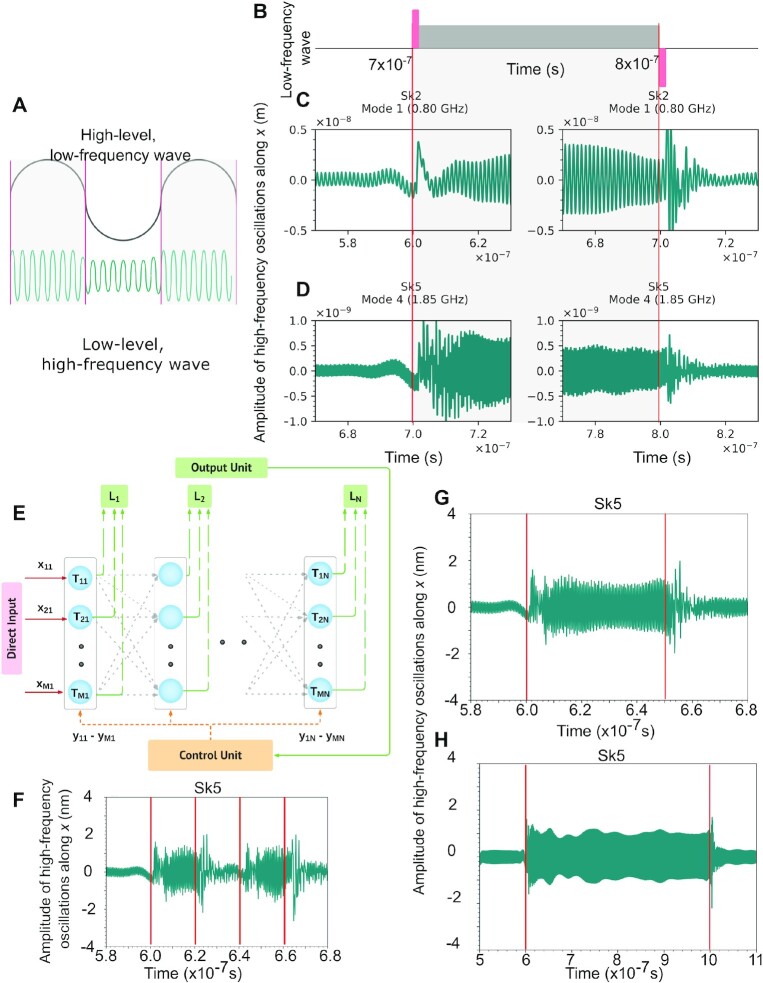
CFC and flexible computing using T-SKONE. (A) Schematic of CFC in the brain. (B to D) CFC implemented with T-SKONE. (B) Schematic of the low-frequency modulatory wave (dark gray) comprising the reconfiguring current pulses (red) and fed via the control input of T-SKONE. (C and D) The high-frequency oscillatory output of the neuron as the latter is reconfigured from Configuration I to II to I, for a driving frequency of (C) 0.80 GHz and (D) 1.85 GHz. (E) Design of a structurally flexible ANN using T-SKONE with N layers comprising M neurons each. Direct input (*x_ij_*), control input (*y_ij_*), and output signals are shown in magenta, orange, and green lines, respectively. The control input can selectively switch every neuron (*T_ij_*) on or off, changing the topology of the network. (A and E) is created in Lucidchart [134]. (F, G, and H) CFC for a low-frequency control signal of varying frequency, i.e. 50, 20, and 2.5 MHz, respectively, and a high-frequency driving input of 1.85 GHz. Reconfiguring pulse times are shown with red lines.

CFC is a cognitive mechanism in which a global, low-frequency neural oscillation *controls or modulates* local, high-frequency neural oscillations in the brain (see Fig. 6A). Involved in three cognitive operations: (i) multi-item representation, (ii) long-distance communication, and (iii) stimulus parsing (21), CFC enables the brain to alter the architecture of its NNs, coordinate message transfer between different NNs, and integrate information across several time scales (32). Here, CFC is realized by feeding a low-frequency wave into the control input of T-SKONE (see Fig. 6B) such that the phase of this wave modulates the amplitude of the high-frequency neuronal output.

CFC is demonstrated using micromagnetic simulations for two cases. In both cases, the control input was a low-frequency modulatory wave of 10 MHz. The direct input fed to T-SKONE was a high-frequency sinusoidal magnetic field with frequency 0.80 GHz in case 1 and 1.85 GHz in case 2. The neuron’s output was taken as the amplitude of oscillation of *Sk2* along *x* for case 1, and that of *Sk5* along *x* for case 2. Here, the amplitude of skyrmion core oscillation is taken as the effective output since it is easy to calculate while being related to the change in magnetization in the nanotrack ($\Delta M)$, the latter being the effective output taken previously. Reconfiguration was carried out by applying an electric current pulse of density 10^11^ A/m^2^ and duration of 0.8 ns using a spin Hall angle of 0.02 corresponding to Pt. Fig. 6C and D plots the output oscillations of *Sk2* and *Sk5* along *x*, respectively, and clearly demonstrate that the low-frequency modulatory input controls the amplitude of the high-frequency neuronal output, implementing CFC.

Taking inspiration from the brain, we present the design of a structurally flexible ANN constructed from T-SKONEs that utilizes CFC to dynamically alter the network architecture. Structurally flexible networks have been previously proposed in software-based ANNs using the techniques of growing and pruning (104). Hardware-based flexible ANNs have achieved structural flexibility by incorporating switches into the network circuit that can switch neurons on or off to alter the network topology (70). Here, we utilize CFC to dynamically turn the adaptive neuron on or off via a control signal without the need for extra switches in the network circuit.

The proposed ANN is feed-forward, fully connected, and consists of *N* layers, with each layer comprising *M* T-SKONEs. A neuron (*T_ij_*) receives a direct input (*x_ij_*), shown in Fig. 6E in gray lines, encoded as above in a sinusoidal magnetic field with frequency 0.80 GHz and carrying information from the dataset or the synaptic layer. The neuron also receives a control input (*y_ij_*), shown in Fig. 6E in orange lines, encoded in modulatory currents that reconfigure T-SKONE and alter its state. The control input *y_ij_ *= 0 (1) corresponds to *T_ij_* in Configuration I (II) with its state off (on). Thus, the control signals can be selected to vary the number of hidden layers from 0 to N-2 and vary the number of neurons in every layer from 1 to M. Input to the ANN is fed to the first layer (plotted with magenta lines in Fig. 6E) and outputs from all neurons are sent to the output unit. The effective output layer of the ANN is the rightmost layer that gives non-zero outputs.

Network topology can be selected by pre-defined execution-control policies (105) or can be dynamically tuned by a control unit (shown in orange in Fig. 6E), which monitors the output of the network and varies network topology by selecting the control signals (*y_ij_*). The network can be trained end-to-end by the stochastic gradient descent method or trained layer by layer. Both techniques of growing and pruning (104) can be implemented. To implement growing, the network is initialized to a minimal layer configuration, trained, tested, and additional layers are added to enhance the accuracy. To implement pruning, the network is initialized to the largest size and is progressively pruned by the removal of neurons until the desired accuracy is reached with the smallest network size. An implementation of network pruning is discussed in Supplementary Material S9 and Fig. S9. The frequency of the high-level control input can be varied in a broad range. Fig. 6C and D shows that after reconfiguration, the neuron adapts to its new state within 20 ns, after which the neuron can be switched to a different configuration. As a result, the control input can adopt frequency values ranging from 50 MHz to >0 Hz. Fig. 6F, G, and H demonstrates CFC for control signal of varying frequencies, including 50, 20, and 2.5 MHz, respectively, while using a driving input of frequency 1.85 GHz.

### Fundamental benefits of T-SKONE-based reconfigurable ANNs

#### Broader AI

Flexible networks provide a general problem-solving tool that can be reconfigured to implement a variety of tasks (70).

#### Compact architecture and energy-efficiency

Flexible networks can be pruned to minimize the network size (104), making the ANN compact and energy-efficient.

#### Fault-tolerant

Flexible networks can be easily tested, and their faults can be isolated (70).

Therefore, structurally flexible networks built using T-SKONE can mitigate multiple challenges facing conventional ANNs, including their narrow intelligence (24) and their large energy and area footprint (82).

### Demonstration of feature-binding using T-SKONE

**Fig. 7.**
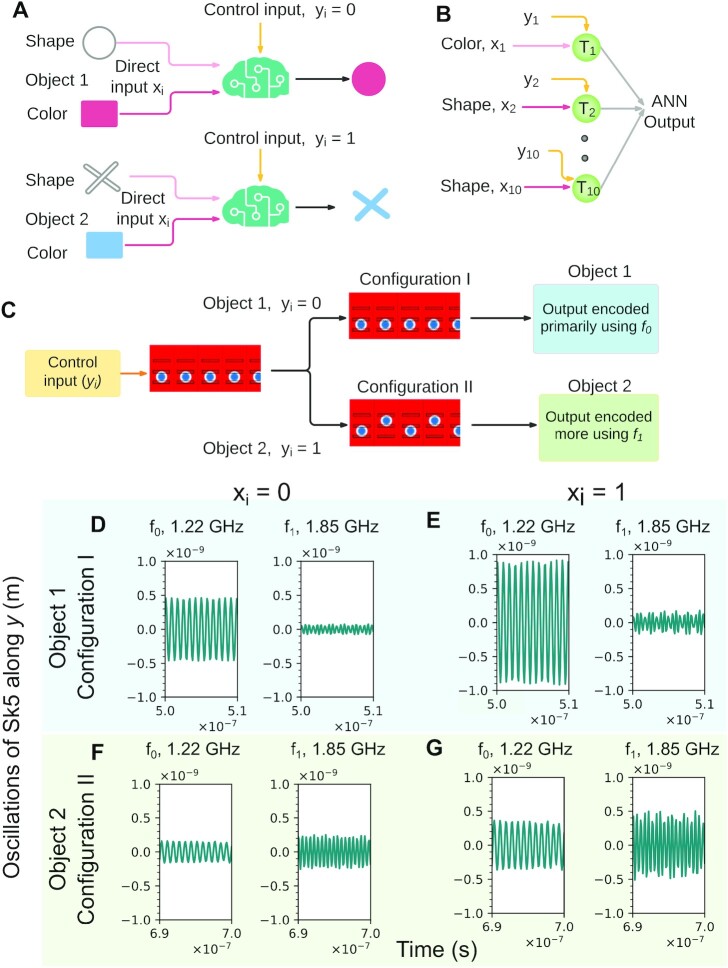
Neuromorphic implementation of feature binding. (A) Schematic of the feature binding. An intelligent agent combines two features, shape and color, to form a coherent percept. (B) Diagram of the adaptive ANN, which consists of one layer of 10 neurons (*T_i_*). Object features are encoded in direct input (*x_i_*), with color encoded in *x_1_* and shape in *x_2_*–*x_10_*. (C) Flowchart of the feature binding process. (D to G) Plot of the oscillations of *Sk5* along *y* with the direct input (*x_i_*) carried by the sum of two sinusoidal magnetic fields of frequencies ${f_0}$ (1.22 GHz) and ${f_1}$ (1.85 GHz). Each subplot shows the neuronal output resolved into frequency components ${f_0}$ and ${f_1}$. Input *x_i_* = 0 is encoded by a magnetic field amplitude of $2.5\;Oe$ and *x_i_* = 1 is encoded by an amplitude of $5.0\;Oe$. (D and E) Neuronal output for object 1 (*y_i_* = 0); the neuron is in Configuration I and the output is predominantly encoded in frequency ${f_0}$. (F and G) Neuronal output for object 2 (*y_i_* = 1); the neuron is in Configuration II and the output is encoded to a greater extent in frequency ${f_1}$.

Feature binding enables the brain to combine different features of a perceived object into a coherent entity (see Fig. 7A) and integrate various fragments of sensory information to build a coherent representation of the outside world (15). Feature binding is known to be important for visual cognition (22) and is related to consciousness (23). Taking inspiration from the brain (16–21), here feature binding is implemented by feeding a low-frequency wave into the control input of T-SKONE such that the phase of this wave modulates the frequency of the high-frequency neuronal output.

In this task, a network of T-SKONEs (see Fig. 7B) receives visual information about two objects, a “magenta circle” and a “blue cross,” in sequence and 100 ns apart. The two object features are color and shape. These features are input separately into an ANN that subsequently combines the two features to build a coherent representation of the original object. This ANN comprises a single layer of 10 T-SKONEs (*T_i_*) that process information in parallel. Through the direct input (*x_i_*) encoded in the amplitude of an oscillating magnetic field, one neuron (*T*_1_) is fed information about the object color, while nine neurons (*T*_2_–*T*_10_) are fed information about the object shape (see Fig. 7B). The control input (*y_i_*) is a low-frequency modulatory wave of 10 MHz. The frequency of this modulatory wave is chosen to enable the network to process objects perceived 100 ns apart. Micromagnetic simulations show that as the phase of the modulatory wave changes from 0 to π, all neurons are reconfigured from Configuration I to II (see Fig. 7C), which changes the frequencies at which the object features are encoded. Details about the simulations are given in Supplementary Material S1.

Fig. 7D to G plots the frequency resolved output characteristics of any one of the 10 identical T-SKONEs that make up the ANN. These characteristics are valid for any *T_i_*, from *T*_1_–*T*_10_. Fig. 7D and E plot the neuronal output when *x_i_* = 0 (*x_i_* = 1) and *y_i_* = 0. In this case, the neuron will be in Configuration I and it will be processing the features of object 1. As can be seen from the plots, object 1 would be encoded primarily at 1.22 GHz, since 1.22 GHz is the predominant output frequency for Configuration I. In contrast, Fig. 7F and G plots the output for *x_i_* = 0 (*x_i_* = 1) and *y_i_* = 1. In this case, the neuron will be in Configuration II and it will be processing the features of object 2. As can be seen from the plots, object 2 would be encoded to a greater extent at 1.85 GHz.

Therefore, the “magenta circle,” which is perceived first, is encoded by the ANN primarily in frequency ${f_0}$ (1.22 GHz) as the neurons are in Configuration I at the time of perception (see Fig. 7D and E). Alternatively, the “blue cross,” which is perceived 100 ns later, is encoded to a greater extent in frequency ${f_1}$ (1.85 GHz) than ${f_0}$ as the neurons are in Configuration II (see Fig. 7F and G). Therefore, the features of the same object are encoded at the same frequency, while features of different objects are encoded at different frequencies by the ANN. These object features can be automatically combined in hardware via synchronization to realize feature binding. Using additional circuitry to filter out the frequency component with a smaller amplitude will encode the first object purely at ${f_0}$ and the second object purely at ${f_1}$, making the ANN more robust.

The two-object perception problem presented here can be further extended to multiobject perception using more resonant frequencies of the T-SKONE. In principle, the maximum number of perceptible objects would be equal to the total number of resonant frequencies of all possible T-SKONE configurations. Additionally, the time difference between perceived objects can be made as small as 0.8 ns, the latter being time required to switch a neuron’s configuration.

Now, we discuss the prospect of experimental implementation of T-SKONE and the device stability, adaptability, and variations involved. The keys to successful experimental implementation of the T-SKONE are (1) precise positioning of skyrmions in the lattice, and (2) consistent, reproducible skyrmion dynamics. The former requires artificial skyrmion pinning sites on magnetic thin films, which can be theoretically achieved by locally modified magnetic parameters (85) or defects (106). Experimentally, it is shown in (107) that skyrmions are effectively pinned by Co atom clusters. The latter depends on well-controlled magnetic material parameters, such as saturation magnetization, magnetic anisotropy, and Dzyaloshinski–Moriya interaction, during skyrmion material growth. With recent advances in voltage control of magnetism [review in (108)], the variation of these parameters in as-grown materials can be compensated. The thermal stability of operation of T-SKONE and the repeatability of its operation under device-to-device variability is demonstrated via simulations in Supplementary Material S10 and Fig. S10. Similarly, Supplementary Material S11 and Fig. S11 show the rapid and robust adaptability of this device. Together these considerations point to successful implementation of T-SKONE in the real-world.

## Discussion

In conclusion, this work proposes the design of a previously unachieved, hardware-based adaptive neuron. This neuron is subsequently utilized to realize three cognitive abilities, namely, context-awareness, cross-frequency coupling, and feature binding. Context-awareness and cross-frequency coupling have not yet been realized in hardware-based neurons. Additionally, the proposed neuron is used to construct an adaptive NN and perform context-aware diagnosis of breast cancer. Our simulations show that the adaptive ANN achieves diagnosis with higher accuracy while learning faster and using a more compact and energy-efficient network than the ANNs that use nonadaptive neurons. The work further describes how hardware-based adaptive neurons like T-SKONE can mitigate several important challenges facing contemporary ANNs by enabling faster learning, compact architectures, broader AI applications, energy-efficiency, and fault-tolerance. However, realizing the potential of T-SKONE requires further development of spin-wave-based synapses and methods for wafer-scale integration. Additionally, designing adaptive neurons where contextual inputs can acquire continuous values and the nonlinearity of the transfer function can be tuned over a wide range will be significant steps forward.

## Supplementary Material

pgac206_Supplemental_File

## Data Availability

All data needed to evaluate the conclusions in the paper are present in the paper and/or the Supplementary Material. The code used to conduct NN simulations is available at (109).
