# Supplementary material for: Adaptive cognition implemented with a context-aware and flexible neuron for next-generation artificial intelligence
Source: PNAS Nexus. 2022 Sep 29;1(5):pgac206. doi: 10.1093/pnasnexus/pgac206 (PMC9802372; doi:10.1093/pnasnexus/pgac206)
Supplement: pgac206_Supplemental_File [file pgac206_supplemental_file.docx]

Supplementary Materials for

**Adaptive cognition implemented with a context-aware and flexible neuron for next-generation artificial intelligence**

Priyamvada Jadaun^1*†^, Can Cui^1†^, Sam Liu^1^ and Jean Anne C. Incorvia^1*^

*Corresponding author. Email: priyamvada@utexas.edu (PJ); incorvia@austin.utexas.edu (JACI)

**This PDF file includes:**

Supplementary Text

Figs. S1 to S12

Table S1

Supplementary Text

S1. Materials and Methods

**Material parameters for micromagnetic modeling** - Material parameters used for micromagnetic modeling of the TmIG/Pt system were taken from references [1] [2]: saturation magnetization $M_{sat}=50\times{10}^{3} A/m$, exchange stiffness $A_{ex}=0.8\times{10}^{-12} J/m$, interfacial Dzyaloshinskii-Moriya constant $D_{ind}=50\times{10}^{-6} J/m^{2}$ , Gilbert damping constant $\alpha=0.02$, perpendicular magnetic anisotropy (PMA) $K_{u}=1.8\times{10}^{3} J/m^{3}$ and $2.0\times{10}^{3} J/m^{3}$ for the reduced anisotropy regions (pinning sites) and normal regions, respectively. The neuron consists of five nanotracks of width of 180 nm (along *x*, coordinates defined in Fig. 1). There were two high-anisotropy track regions of length 56 nm (along *y*) alternating between three low-anisotropy pinning wells of length 12 nm (along *y*). The neuron was padded along *x* and *y* with unpatterned magnetic material to enhance skyrmion stability and closed boundary conditions were employed. In all simulations, a DC magnetic field of amplitude $H_{DC}=240 Oe$ was applied to the neuron in order to stabilize the skyrmions. The application of this DC field is required for skyrmion stability specifically in the TmIG/Pt system [1], but is not required for the neuron’s design or functionality.

**Simulation of the FFT spectrum** - To calculate the FFT spectrum, the neuron was first excited with an external magnetic field $H_{RF}=H_{0}sinc(\omega t)$ along *x* for 1 ns, with the amplitude of the magnetic field $H_{0}=40 Oe$ and the cutoff frequency $\omega=2\pi\times f$ where $f$ = 60 GHz. The field was applied to all nanotracks for 1 ns. The neuron was subsequently allowed to relax, and its dynamics during relaxation was examined and the trajectories of the skyrmion cores were calculated using the method described in [3].

**Amplitude Modulation** - To demonstrate Amplitude Modulation, T-SKONE was excited in all nanotracks with a sinusoidal magnetic field $H_{RF}=H_{0}sin(\omega t)$ along *x* with $H_{0}=10 Oe$ and $\omega=2\pi\times f$, at a range of driving frequencies $f$, and the oscillatory output was calculated. The oscillations of the skyrmion cores along *x* and *y* are taken to be the neuronal response.

**Micromagnetic simulations to demonstrate feature-binding** - Feature binding is a cognitive ability of the brain by which it combines different features of a perceived object that are processed in different regions of the brain to form a coherent perception of the entire object. Taking inspiration from the brain, we implement feature binding based on synchrony and phase-coding. Therein, the phase of a low-frequency modulatory oscillation helps to bind the various features of an object by ensuring that different features of the same object are encoded at the same frequency, such that they can be correctly combined through synchronization.

In this task, a network of 10 T-SKONEs was designed to receive visual information about two objects, a ‘red circle’ and a ‘blue cross’, in sequence and 100 ns apart. The two object features are color and shape. The ANN was designed as a single layer network of 10 T-SKONEs (*T_i_*) such that one neuron (*T_1_*) processes information about the object color, while nine neurons (*T_2_*-*T_10_*) processes information about the object shape, in parallel.

Information about the object features was fed as direct input (*x_i_*) to the neurons and encoded in the amplitude of an oscillating magnetic field $H_{RF,i}=H_{0,i}(sin\left( \omega_{0}t \right)+sin\left( \omega_{1}t \right))$, where $\omega_{j}=2\pi\times f_{j}$ and $f_{0}= 1.22$ GHz and $f_{1}=1.85$ GHz. The color ‘red’ (‘blue’) corresponded to *x_1_* = 0 (1) and was input to neuron *T_1_*. The shape of the objects was in the form of a 3×3 pixel array, where ‘off’ (‘on’) pixel states corresponded to *x_i_* = 0 (1), for i = 2-10. Thus, the ‘circle’ was represented with an input vector {x_2_, x_3_,.., x_10_} = {1, 1, 1, 1, 0, 1, 1, 1, 1}, and the ‘cross’ was represented with a vector {x_2_, x_3_,.., x_10_} = {1, 0, 1, 0, 1, 0, 1, 0, 1}. The direct input for every neuron, *x_i_* = 0 (1) corresponded to magnetic field amplitude $H_{0,i}=2.5 Oe (5.0 Oe)$. In a hardware implementation of this task, the visual information regarding object features could perceived by a retinomorphic sensor [4, 5] and converted by a waveform generator into an oscillating magnetic field which would be fed as direct input (*x_i_*) to the T-SKONE layer.

The control input (*y_i_*) was a low-frequency modulatory wave of 10 MHz which constituted modulatory currents *J_2_* and *J_4_*, such that all neurons (*T_i_*) switched their configurations every 100 ns, thereby switching their output frequencies. Thus, objects perceived in different time windows were encoded at different frequencies. Micromagnetic simulations were conducted for a T-SKONE in the ANN described above, and the effective output of the neuron was taken to be the oscillatory output of *Sk5* along *y*. This oscillatory output was filtered to obtain the two frequency components, i.e., $f_{0}= 1.22$ GHz and $f_{1}=1.85$ GHz.

S2. Amplitude Modulation in T-SKONE along *y*

To demonstrate Amplitude Modulation, the neuron was excited by a sinusoidal magnetic field for a range of frequencies for both configurations and the resulting neuronal dynamics was analyzed. Figure S1 (A-D) shows the amplitudes of oscillations of the skyrmion cores along *y* at driving frequency ranges centered on (A) Mode 1, (B) Mode 2, (C) Mode 3, and (D) Mode 4. The plots show multiple cases of differences in the amplitudes of skyrmion oscillations between Configurations I (solid blue) and II (dashed magenta), thereby demonstrating Amplitude Modulation along *y*. This is analogous to Fig. 2 (C-F) which show Amplitude Modulation along *x*.

S3. Resonant modes of T-SKONE in Configuration II

Figure S2 plots the resonant modes of T-SKONE in Configuration II, when the neuron is excited by a sinusoidal magnetic field. This is analogous to Fig. 3 which plots the resonant modes of T-SKONE while it is in Configuration I. The first and second columns of Fig. S2 (A-D) plot the oscillations for every skyrmion core along *x* and *y*, respectively, for resonant modes (1-4). Similar to Configuration I, the skyrmion cores oscillate sinusoidally with frequencies locked to the driving input and with a variety of amplitudes. The topological charge density maps of T-SKONE are plotted at times $t = 0$, $T/4$, $T/2$ and $3T/4$, with $T$ being the respective time period of oscillation, to investigate the physical origin of these modes. These maps are shown in the four columns on the right of Fig. S2. Similar to the dynamics seen for Configuration I, the four modes show distinct origin including skyrmion breathing (Mode 1), hybridization of breathing and counterclockwise (CCW) gyration (Mode 2), pure CCW gyration (Mode 3) and hybridization of breathing, CCW and clockwise gyration (Mode 4). Once again, the oscillation amplitudes are generally larger for the outer skyrmions (*Sk1* and *Sk5*) than amplitudes for inner skyrmions (*Sk2* - *Sk4*).

S4. Coupled Skyrmion Gyrotropic Modes in Metallic Ferromagnetic System

The heavy metal (HM)/CoFeB/metal oxide (MO_x_) trilayer stack exhibiting perpendicular magnetic anisotropy (PMA) and Dzyaloshinskii-Moriya interaction (DMI) has been demonstrated in experiments to allow for the creation, manipulation and detection of room-temperature skyrmions [6]. We again use anisotropy-based pinning sites to precisely position the skyrmions in the lattice. Fig. S3 (A) shows the double pinning sites design and a relaxed skyrmion positioned in lattice site A. As visible, the skyrmion has two opposite edges located within the pinning sites and is slightly elongated along *y* due to the pinning effect.

Individual or coupled skyrmions are known to demonstrate a gyrotropic mode when excited with an in-plane magnetic field [7] [8]. We first determine gyrotropic oscillation frequency of an isolated skyrmion in the artificial lattice site (see Methods). The fast Fourier transform (FFT) power density spectrum Fig. S3 (B) shows a single peak at $f_{0}$= 0.66 GHz. To identify the nature of this resonant oscillatory mode, the skyrmion was excited with a sinusoidal field $H_{y}\left( t \right)=H_{0}sin(\omega t)$ with $\omega=2\pi\times f_{0}$ in *y* for a duration $t =$ 50 ns. The trajectory of the skyrmion core position (*x*, *y*) plotted in Fig. S3 (C) reveals a clockwise (CW) gyrotropic mode, with resonant amplification indicated by the increasing oscillation amplitude with time. The oscillation amplitude saturates at $D_{x}=$ 5.6 nm and $D_{y}=$ 3.8 nm, which can be attributed to skyrmion edge repulsion and pinning effects. In contrast, when the input oscillatory field is off-resonant with frequency $f_{0}$= 0.70 GHz, the oscillation amplitude is significantly lower with $D_{x}$ = 1.4 nm and $D_{y}$ = 0.8 nm.

Resonance spectra of five coupled skyrmions in lattice Configurations I and II are next simulated, with FFT power density spectra of skyrmion core oscillations shown in Fig. S4 (A-B). Five resonance frequencies can be identified in both configurations: for Configuration I, five resonant frequencies are found to be $f_{1}$ = 0.35 GHz, $f_{2}$ = 0.49 GHz, $f_{3}$ = 0.65 GHz, $f_{4}$ = 0.80 GHz and $f_{5}$ = 0.90 GHz; for Configuration II, the five resonant frequencies shifted to $f_{1}^{'}$= 0.38 GHz, $f_{2}^{'}$ = 0.49 GHz, $f_{3}^{'}$ = 0.61 GHz, $f_{4}^{'}$ = 0.71 GHz and $f_{5}^{'}$ = 0.76 GHz. The shift in resonant frequencies between Configurations I and II demonstrates Frequency Modulation.

Amplitude Modulation is demonstrated by exciting the lattice with a driving field at the resonant frequencies $f_{1}$-$f_{5}$ of Configuration I. The oscillation amplitudes of the skyrmion cores are compared for both Configurations I and II, shown in Fig. S4 (C). As notable, except for $f_{2}$ and $f_{2}^{'}$, the skyrmion oscillation amplitudes of Configuration II are reduced as compared to Configuration I because the system is out of resonance. Since $f_{2}^{'}\approx f_{2}$, no substantial change of oscillation amplitude is observed for that particular frequency.

The difference between the five gyrotropic frequencies lies mainly in the phase relations of the gyrotropic motion between each skyrmion. In Fig. S4 (D), skyrmion lattice is in Configuration I and are resonantly excited at frequencies $f_{1}$-$f_{5}$ and the oscillations along *x* of skyrmion cores from time $t =$ 30 ns to $t =$ 40 ns are plotted. To maintain the phase relations between each skyrmion, in the simulation, oscillatory input field was applied to only one skyrmion at each frequency: for frequencies$f_{1}$, $f_{3}$, and $f_{5}$, *Sk3* is driven; for frequencies $f_{2}$and$f_{4}$, *Sk2* is driven. All skyrmions still have CW gyrations, but each gyrotropic frequency exhibits different phase relations between individual skyrmions. As shown in Fig. S3(D), we have obtained the phase relationships between the skyrmions by directly exciting the skyrmions, and the result is in agreement with previous reports in literature [6].

S5. Construction of the multi-modal dataset for breast cancer diagnosis

In this task, breast cancer diagnosis is performed by an ANN that classifies biopsy image features of breast mass into benign and malignant categories. In addition to direct information that comprises biopsy image features, the ANN also uses contextual information comprising patient medical data to perform the diagnostic prediction.

Owing to the lack of a well-known, multi-modal cancer dataset containing information about both biopsy images and patient medical history, we constructed such a dataset by appending the Breast Cancer Wisconsin (Diagnostic) dataset (comprising biopsy image features) [9] with information regarding patient medical data (comprising attributes *𝛂_i_*). The modified dataset was constructed so as to capture the statistical correlations reported in literature between the attributes or risk factors and the presence of cancer. For instance, the dataset replicated the real-world situation where breast cancer was 100 times more likely for women than for men. This dataset was obtained by using Bayesian inference, the details of which are described in Supplementary Information S4. It is worthwhile to note that in real-world applications, information about attributes for a patient should be easily available from their medical history. This method can be extended to predict malignancy from mammographic elements for early cancer screening [10].

Patient medical data comprises well-known risk factors for breast cancer and was used as contextual input that provides enhanced information about a patient’s health to the neural network. Four risk factors for breast cancer were selected. For every factor (labeled attribute *𝛂_i_*), we found information from literature about the risk of getting breast cancer given that a patient has attribute *𝛂_i_* vs. the risk of getting breast cancer if that patient does not have attribute *𝛂_i_*. This is labeled relative risk (*u_i_*). The attributes selected for this study are described below.

1. **Patient is female** - The risk for breast cancer in America is 100 times higher if the patient is female rather than male [11].
2. **Age of the patient is greater than 50 years** - Age is one of the most important risk factors of breast cancer, as the risk for developing cancer is known to be 1/53 for age < 49 years, 1/43 for 50< age <59 years, 1/23 for 60 < age <69 and 1/15 for age >70 across the world [12].
3. **Patient has a Body Mass Index (BMI) in the obese range** - Women with a BMI in the obese range (BMI > 30) have a threefold increased risk of breast cancer, as reported by a study conducted in Iran [13].
4. **Alcohol intake of patient is high** – In data collected worldwide, women with a high alcohol intake (of at least 27 units per week) were 51% more likely to develop breast cancer as compared to non-drinkers [11].

These four binary attributes were treated as independent variables and formed the four contextual inputs, such that every attribute (e.g. patient is female) was associated with ‘True’ or ‘False’ values. This data was fed to the ANN as contextual information to perform the cancer diagnosis.

Here, we derive a multi-modal dataset for breast cancer diagnosis by augmenting the Breast Cancer Wisconsin (Diagnostic) dataset (comprising biopsy image features) with information regarding patient medical data (comprising attributes *𝛂_i_*). The four selected attributes are:

1. Patient is female
2. Age of the patient is greater than 50 years
3. Patient has a Body Mass Index (BMI) in the obese range
4. Alcohol intake of patient is high

These attributes are binary variables with ‘True’ or ‘False’ values and are assumed to be independent, thus a patient in the dataset can be assigned a value for one attribute (*𝛂_i_*) regardless of the value assigned to the patient for a different attribute (*𝛂_j_, j≠i*). The risk of a patient having cancer given that attribute *𝛂_i_* is ‘True’ vs. the risk of that patient having cancer given that *𝛂_i_* is ‘False’, is termed as relative risk (*u_i_*). To ensure that the multi-modal dataset is representative of reality, *u_i_* is obtained from literature for all *𝛂_i_*. The goal is to calculate the number of patients in the dataset that would have *𝛂_i_* = ‘True’ such that the dataset captures real-world statistics for breast cancer.

Let *A_i_* be the event that a patient in the dataset has attribute *𝛂_i_*, = ‘True’. Thus, P(*A_i_*) is the probability of this event. Therefore, the probability that a patient in the dataset has attribute *𝛂_i_* = ‘False’ is $P\left( A_{i}^{'} \right)=1-P\left( A_{i} \right)$. The number of patients in the dataset with attribute *𝛂_i_* = ‘True’ is $P\left( A_{i} \right)N$, where $N$ is the total number of cases in the dataset. Number of patients in the data set with attribute *𝛂_i_* = ‘False’ is $(1- P\left( A_{i} \right))N$. In the Wisconsin (Diagnostic) dataset, *N* = 569, with number of cases with cancer (*N_cancer_*) = 212 and number of cases without cancer (*N_benign_*) = 357. Let B be the event that a patient in the dataset has cancer. Therefore, $P\left( B \right)=\frac{212}{569}\equiv v, P\left( B^{'} \right)=\frac{357}{569}=1-v$.

We would like to find the probability that a patient has attribute *𝛂_i_* = ‘True’, given that they have cancer, which is the conditional probability $P\left( A_{i} | B \right).$ Then, from amongst the patients in the dataset who are known to have cancer, $P\left( A_{i} | B \right)N_{cancer}$ cases can be assigned with *𝛂_i_* = ‘True’ and $(1-P\left( A_{i} | B \right){)N}_{cancer}$cases can be assigned with *𝛂_i_* = ‘False’. Similarly, we need to calculate the probability that a patient has attribute *𝛂_i_* = ‘True’, given that they do not have cancer, which is the conditional probability $P\left( A_{i} | B^{'} \right)$. Subsequently, from amongst the patients in the dataset who do not have cancer, $P\left( A_{i} | B^{'} \right)N_{benign}$ cases can be assigned with *𝛂_i_* = ‘True’ and $(1-P\left( A_{i} | B^{'} \right){)N}_{bening}$cases can be assigned with *𝛂_i_* = ‘False’, which accomplishes the task of augmenting the dataset with attribute values.

The relative risk for a given attribute $u_{i}=\frac{P\left( B | A_{i} \right)}{P\left( B | A_{i}^{'} \right)}$.

In general, $P\left( B | A_{i} \right)$ which is the probability that a patient in the data set has cancer, given that they have attribute *𝛂_i_* is unknown to us (except in the case of obesity). This is because the Wisconsin dataset is not representative of the general population and the patients in the dataset have been selected based on some prior risk analysis whose statistics is unknown to us. Therefore, $P\left( B | A_{i} \right)$ is labeled *x_i_* and is an independent variable in this analysis that is tuned to reasonable values. Consequently, $P\left( B | {A_{i}}^{'} \right)=\frac{x_{i}}{u_{i}}$*.*

**Derivation of** $\boldsymbol{P}\left( \boldsymbol{A}_{\boldsymbol{i}} | \boldsymbol{B} \right)$**:**

Using Baye’s Theorem and the identity $P\left( A_{i} | B \right)+ P\left( {A_{i}}^{'} | B \right)=1$, we obtain:

$$\frac{P\left( B | A_{i} \right)*P\left( A_{i} \right)}{P\left( B \right)}+ \frac{P\left( B | {A_{i}}^{'} \right)*P\left( {A_{i}}^{'} \right)}{P\left( B \right)}=1$$

$$\frac{x_{i}*P\left( A_{i} \right)}{v}+ \frac{x_{i}*(1-P\left( A_{i} \right))}{u_{i}v}=1$$

$$P\left( A_{i} \right)= \frac{u_{i}v-x_{i}}{x_{i}(u_{i}-1)}$$

Therefore, $P\left( A_{i} | B \right)=\frac{P\left( B | A_{i} \right)*P\left( A_{i} \right)}{P\left( B \right)}= \frac{x_{i}*P\left( A_{i} \right)}{v}$ and $P\left( A_{i}' | B \right)=1-P\left( A_{i} | B \right)$

**Derivation of** $\boldsymbol{P}\left( \boldsymbol{A}_{\boldsymbol{i}} | \boldsymbol{B}^{\boldsymbol{'}} \right)$**:**

$$P\left( A_{i} | B^{'} \right)= \frac{P\left( B^{'} | A_{i} \right)*P(A_{i})}{P(B')}$$

Therefore, $P\left( A_{i} | B^{'} \right)= \frac{(1-P\left( B | A_{i} \right))*P(A_{i})}{P(B')}= \frac{(1-x_{i})*P(A_{i})}{(1-v)}$ and $P\left( A_{i}' | B^{'} \right)=1-P\left( A_{i} | B^{'} \right)$

The final distribution of the attribute values are shown in Table S1.

S6. Adaptive neuron responds to a dynamic environment in human-machine interaction task

Here, we demonstrate the ability of T-SKONE to adapt its response in an environment where environmental safety is changeable and uncertain. The task chosen is a human-machine interaction task, where the neuron receives a direct input representing a human spoken command and a control input representing environmental safety, specifically whether a box is safe to open. Upon receiving a ‘go’ command from the human being, the neuron can adaptively decide whether or not to open the box, depending on whether the box is safe or not.

This task was implemented using micromagnetic simulations. Here, the spoken human command was represented by the direct input and the color of the box was represented by the control input, with the latter modulating the configuration of T-SKONE. This task was designed such that the human command would be sensed by a neuromorphic acoustic sensor [14] (represented in Fig. S6 (A) by a layer of yellow circles), that converts a human speech signal into an oscillatory voltage signal decomposed into different frequency channels in the range 1 – 15 kHz. Using frequency mixing, these signals could be combined with sinusoidal signals in the GHz range, resulting in AC current of various frequencies that can excite T-SKONE dynamics. As a simple example, here we selected two frequencies namely 1.22 GHz and 1.85 GHz, such that, the command to refrain from opening the box was represented by a sinusoidal magnetic field of frequency 1.22 GHz (labeled direct input ‘0’), whereas that to open the box was represented by frequency 1.85 GHz (labeled direct input ‘1’). The amplitude of the sinusoidal magnetic field was $10 Oe$. Additionally, the color of the box was designed to be detected by a neuron in the retinomorphic sensor layer [4, 5], (shown in Fig. S6 (A) by a blue circle). The digital voltage output of this neuron would encode the contextual input, where the absence of a voltage pulse signified that the box is not red in color (labeled ‘0’) and the presence of a voltage pulse signified that the box is red (labeled ‘1’). This voltage pulse would give rise to modulatory current inputs $J_{2}$ and $J_{4}$ (i.e., currents that reconfigure the second and fourth skyrmions along *x* in Fig 1 (A)), such that T-SKONE which was initially in Configuration I, upon receiving the voltage pulse switched to Configuration II. The dynamical response of T-SKONE, which depends on the collective action of the two inputs, was simulated for all four combinations of inputs, and the output oscillations of *Sk5* were analyzed (see Fig. S6 (C-F)). If the amplitude of oscillations of *Sk5* along *y* were smaller than 1 nm, then the neuron had decided to open the box.

As shown in Fig. S6 (B, C-F), the neuron correctly decides to open the box, only under the conditions that it received a “go” command from the human collaborator (direct input ‘1’) and that the box is not red (control input ‘0’). Critically, the solution is designed in such a way that when the box is red in color (Configuration II), T-SKONE will not open the box regardless of the human command, making the latter irrelevant. However, when the box is not red in color (Configuration I), the decision to open the box exactly follows the human command. Therefore, the response of T-SKONE to the human command changes with the environment “on the fly”. The key reason is that in Configuration II the oscillations of *Sk5* along *y* have considerable amplitude for both driving frequencies. However, in Configuration I the amplitude of oscillation remains large at 1.22 GHz but is smaller by a factor of 4 at 1.85 GHz from the amplitude seen in Configuration II.

S7. Study of the context input sequence

Here we present a brief examination of the impact of context input sequence on the prediction accuracy of the context-aware network. For the initial simulation, the contextual inputs are arranged in the sequence (𝜶_1_, 𝜶_2_, 𝜶_3_, 𝜶_4_) and fed into (7, 7, 8, 8) neurons of Layer 1. In subsequent three simulations, the input sequence is chosen to be (𝜶_2_, 𝜶_1_, 𝜶_3_, 𝜶_4_), (𝜶_3_, 𝜶_2_, 𝜶_1_, 𝜶_4_) and (𝜶_4_, 𝜶_2_, 𝜶_3_, 𝜶_1_). The results of these simulations are shown in Fig. S7 and signify that the choice of input sequence impacts the prediction accuracy. Thus, the performance of the context-aware network can be further optimized by tuning the input sequence of contextual attributes.

S8. Comparison of T-SKONE performance with state-of-the-art

For a more thorough benchmarking of the performance of T-SKONE with state-of-the-art non-adaptive neurons, we implement the direct data fusion method (as described in the main text) using a software perceptron and compare it to context-aware diagnosis. Both networks have 34 input neurons, 2 output neurons and 68 synapses. The results of these simulations are shown in Fig. S8.

The software network achieves a final accuracy of 98% while the context-aware network shows a smaller a final accuracy of 97%. It is hypothesized that the reduced performance of the context-aware network is the result of the encoding scheme selected for this network which prohibits the use of negative inputs/outputs, reducing the expressivity of data. For the software neuron, the input is normalized according to the available data (which includes negative values) and the output can be mapped directly to the output, where a softmax activation is performed. This ensures that the network is well-regularized. In contrast, the input and output values for the T-SKONE neuron are restricted to be positive. Thus, the input data for Layer 1 is normalized uniformly to fall between a minimum of 30 Oe and maximum of 45 Oe. As a result, the dataset for T-SKONE is less expressive which is known to be detrimental to network performance [15].

However, this constraint on T-SKONE input/output values is artificial and can be removed by a simple extension of our initial encoding scheme. Along with encoding the absolute value of inputs in the amplitude of the external magnetic field (*H*_RF_), the sign of inputs can be encoded in the phase of *H*_RF_. For instance, if x_i_=1 is encoded in *H*_RF_ = $H_{i}sin(\omega t)$, x_i_=-1 can be encoded in *H*_RF_ = $H_{i}sin(\omega t+\pi)$. This simple extension will allow for positive and negative data values that are represented by AC currents with opposite phases. These currents will innately get added at the synapse level and fed into the next layer with their phases (signs) correctly taken into account.

S9. Utilizing a CFC-based structurally flexible network to implement pruning

To visualize a real-world application of the structurally flexible network described in this work, we consider the implementation of Layer-wise Iterative Pruning [16] which can reduce the size of a network without compromising its predictive ability. In [16], the authors utilized a fully connected, feed-forward network with 3 layers. Layer 1 was the input layer, Layer 2 was the hidden layer with weight matrix W^1^ and Layer 3 was the output layer with weight matrix W^2^. Layer 2 comprised 1600 neurons in the baseline network. This network learnt from the bank marketing dataset obtained from [17] and predicted the success of bank telemarketing.

The baseline network however consisted of several redundant neurons that did not contribute much to the predictions. To solve this, the authors presented two pruning algorithms, where pruning is the process of removing redundant neurons to reduce network size without significantly degrading the prediction accuracy. As an example, if we consider two hidden neurons of the network described above (Neuron 1 & 2, see Fig. S9) then the weight matrix for inputs to these hidden neurons can be written as $W^{1}= \left[ \begin{matrix} W_{11}^{1} & W_{21}^{1} \\ W_{12}^{1} & W_{22}^{1} \end{matrix} \right]$ where $W_{ij}^{1}$corresponds to the weight associated with the i^th^ neuron of Layer 1 and j^th^ neuron of Layer 2. If post-training, the baseline network reaches a state where the two rows of $W^{1}$are identical, then the hidden neurons 1 & 2 will function identically and never produce distinctive outcomes, i.e. the neurons will be redundant. The pruning algorithms in this work aims to identify such redundancies in the baseline network and remove them. To achieve this, the cosine algorithm conducts a row-wise normalization on the targeted weight matrix and calculates the degree of similarity between different neurons (labelled cosine similarity). Neurons with similarity greater than a cut-off are eliminated. The pruned network thus obtained, is retrained on the dataset. The authors report that for the bank marketing dataset, pruning reduced the network size to one-tenth of the baseline while reducing accuracy by 20%. A more sophisticated form of pruning called Taylor expansion pruning reduced the network size to one-tenth only at a cost of 3% accuracy rates.

This application can be implemented with a 3-layer, feed-forward network using T-SKONE by encoding the bank dataset input in the amplitude of the oscillatory magnetic field (*H*_RF_) with a frequency of 0.80 GHz. In the baseline network, the hidden layer would be composed of 1600 neurons each of which is a T-SKONE in Configuration II, i.e., these neurons would be switched ‘on’. This network would be trained on the dataset to predict the success of bank marketing. Post-training, the weights stored in the synapses of Layer 1 would be read and this information would be sent to the Control Unit (CU), which would implement the pruning algorithm on the weight matrix as described in [16]. The CU would identify all redundant neurons in Layer 1 and send a CFC modulatory signal to change the state of these neurons to Configuration I, effectively switching them ‘off’. As a result, the pruned network would be significantly more compact and energy-efficient than before without serious loss in its prediction accuracy.

S10. Demonstration of stability and repeatability of T-SKONE performance for real-world applications

This section demonstrates (i) the thermal stability of operation of T-SKONE and (ii) the repeatability of its operation under device-to-device variability.

To verify the thermal stability of T-SKONE at room temperature, finite-temperature effects were added to the micromagnetic simulations through a randomly fluctuating thermal field [18]. Specifically, this thermal field term was assumed to be a zero-mean Gaussian distribution with standard deviation *B_therm_* described below.

$$B_{therm}= \sqrt{\frac{2\alpha k_{B}T}{\mu_{0}\gamma V_{F}M_{s}\Delta t}}$$

where, α is the damping parameter, *k_B_* is the Boltzmann constant, *T* is the temperature taken to be 300 K, *M*_s_ is the saturation magnetization, γ is the gyromagnetic ratio, *V_F_ is* the device volume, ∆*t* is the time step taken to be 1ns.

Micromagnetic simulations were conducted incorporating this thermal term for 25 samples for Configuration I and II. An external excitation field of 0.8 GHz and 10 *Oe* was applied. Fig. S10 (A) plots the amplitude of oscillations of skyrmion 4 along *x*. As can be seen, while the output values vary with fluctuations in the thermal term, the output of Configuration I and II can be easily distinguished in all 25 samples except 1. Specifically, the output amplitude for Configuration I is always greater than the corresponding amplitude for Configuration II. This demonstrates the thermal stability of the neuron device under real-world conditions.

To verify the repeatability of the device performance, device-to-device variability effects were added to the micromagnetic simulations. For this, the DMI constant and saturation magnetization (*M_s_*) of the TmIG/Pt bilayer used in the device was varied over a range of ±10% to mimic real world conditions. Micromagnetic calculations were carried out using these altered material parameters for both Configuration I and II. An external excitation field of 0.8 GHz and 10 *Oe* was applied. Fig. S10 (B & C) plot the amplitude of oscillations of skyrmion 4 along *x* for variation in DMI & saturation magnetization, respectively. As can be seen, while the output values vary with changes in the material parameters, as expected, the output of Configuration I and II can be easily distinguished in all cases. Specifically, the output amplitude for Configuration I is always greater than the corresponding amplitude for Configuration II. This demonstrates the repeatability of the neuron device performance under real-world conditions.

S11. Demonstration of the adaptability of T-SKONE

This section examines the adaptability of T-SKONE with time and shows it to be rapid and robust.

The simulations previously performed for the cross frequency coupling (CFC) demonstration also show the adaptability of T-SKONE with time as it is switched from Configuration I to II and back. Fig. S11 (A) plots the output amplitude of oscillations of skyrmion 2 along *x* under a constant magnetic field input with frequency 0.8 GHz and amplitude 10 *Oe*. The neuron is switched from Configuration I to II and back, under this constant field input, and the output variation is plotted with time. As can be seen from Fig. S11 (A), as the neuron is switched, it adapts to its new state within 20 ns. The output oscillations of the neuron adjust to the new neuronal state and reach a final stable value in this time. This rapid adaptability of the neuronal output occurs for switching from Configuration I to II as well as from II to I.

The rapid adaptation performed by T-SKONE is further verified by an examination of the decay of output oscillations after the input magnetic field is switched off. Fig. S11 (B) plots the decay with time in output amplitude of oscillations of skyrmion 4 along *x* when excited by an external magnetic field with frequency 0.8 GHz and amplitude 30 *Oe* that is subsequently turned off, both for Configuration I and II. In both cases, the amplitude of oscillation decays rapidly with time, reducing by 50% in value within 10 ns and reaching negligible values by 30 ns. Similar calculations are performed for an external magnetic field with frequency 0.8 GHz and amplitude 42 *Oe* (shown in Fig. S11 (C). Once again, the amplitude of oscillation decays rapidly with time, reducing by 50% in value within 10 ns and reaching negligible values by 30 ns.

These results demonstrate the fast and repeatable adaptability of T-SKONE to varying external conditions.

S12. MNIST digit recognition task performed with adaptive ANN using T-SKONE

To further demonstrate the accuracy of the adaptive ANN and benchmark it with well-established machine learning tasks and large datasets, we include a simulation of the MNIST digit recognition task. Routinely used to benchmark neural networks [19, 20, 21, 22, 23], the MNIST task is demonstrated utilizing the adaptive ANN, a baseline network and a direct-fusion network.

For this simulation the MNIST dataset was used [24], with 60,000 images in the training set and 10,000 images in the validation set. A batch size of 300 was used in all cases. All cases were trained for 15 epochs for 5 different seeds. The network architecture was that of a perceptron with 794 neurons comprising 28*28 input neurons and 10 output neurons, with a softmax activation at the output. The optimizer used was Adam, with an optimized learning rate of 0.01 for all networks.

As before, the baseline network was fed the digit image data (direct input) and was made up of 794 T-SKONE neurons in Configuration I. However, in addition to this image data (direct input), the adaptive and direct-fusion networks were also fed one contextual input which carried information about whether the input digit was odd or even. As before, this contextual information was fed into the adaptive ANN using the modulatory input (*y_i_*) for all input neurons. Specifically, all input neurons were switched to Configuration I (II) for even (odd) inputs. In contrast, for the direct-fusion network the contextual information was fed into an input neuron that adopted a maximum value when the digit was odd and a minimum value when the input was even.

As can be seen from the results (Fig. S12), the adaptive ANN performs the task with as much accuracy as the direct-fusion task (92.5%) while showing faster learning. The small difference in performance of the two networks is understandable as the two networks have very little contextual information to process, thus the adaptive network does not have the opportunity to carry out context-dependent inference. However, the high accuracy of inference by the adaptive ANN is notable as it shows that this network is able to successfully perform complex machine learning tasks with large datasets.

Fig. S1.

FIG. S1. Amplitude modulation along *y*. (A-D) Plots of the amplitudes of oscillation for all five skyrmions along *y* shown at a range of frequencies for Configuration I (blue circles) and Configuration II (magenta triangles).

Fig. S2.

FIG. S2. Resonant modes of T-SKONE in Configuration II when excited by a sinusoidal driving field of frequency (A) 0.80 GHz (Mode 1), (B) 1.04 GHz (Mode 2), (C) 1.22 GHz (Mode 3) and (D) 1.85 GHz (Mode 4). The two left most columns plot the output oscillations along *x* and *y*, respectively. The four columns on the right plot the topological charge densities at times $t = 0$, $T/4$, $T/2$, $3T/4$, where $T$ is the respective time period of oscillation. The color maps for topological charge density ranges from $-1.5\times{10}^{14}m^{-2}$ (magenta) to zero (green).

Fig. S3.

Fig. S3. Gyrotropic mode of a single skyrmion. (A) Simulated skyrmion stabilized in a two-site lattice. Shadowed regions have reduced perpendicular magnetic anisotropy and pin the skyrmion edges. (B) FFT spectra of skyrmion gyrotropic oscillation. Inset: zoom-in to the frequency range 0.5-0.8 GHz. A resonant peak is identified at $f_{0}$= 0.66 GHz. (C)-(D) Calculated core position trajectories of the skyrmion driven by sinusoidal magnetic field with resonant ($f_{0}$= 0.66 GHz) and off-resonant ($f_{0}^{'}$ = 0.70 GHz) frequencies, respectively, for duration $t =$ 50 ns.

Fig. S4.


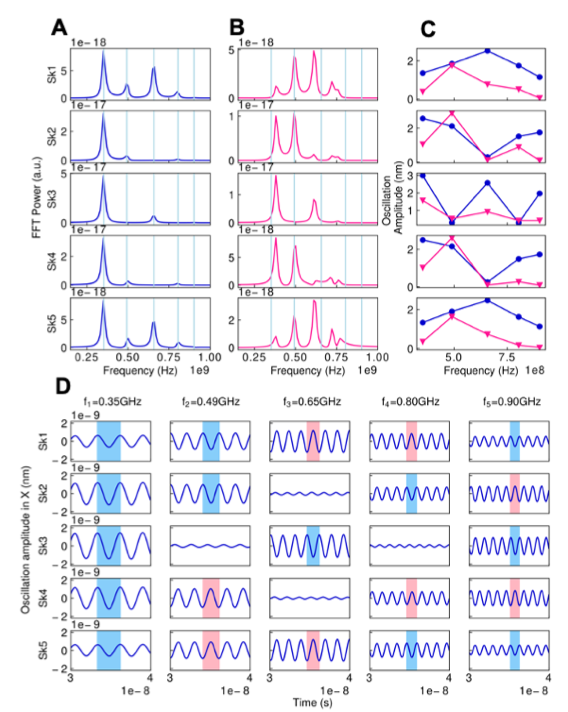


Fig. S4. Coupled gyrotropic dynamics of the reconfigurable skyrmion lattice. (A) and (B) FFT power density spectra of collective skyrmion gyrotropic oscillations, for lattice Configurations I (AAAAA) and II (ABABA), respectively. Resonant frequencies $f_{1}$-$f_{5}$of Configuration I are marked by blue vertical lines. (C) Skyrmion gyrotropic oscillation amplitudes driven at resonant frequencies $f_{1}$-$f_{5}$ of Configuration I, for lattice Configurations I (blue) and II (magenta), respectively. (D) Resonant skyrmion oscillations along *x* of lattice Configuration I; here each column corresponds to a specific driving frequency ($f_{1}$-$f_{5}$) and each row corresponds to a specific neuron (*Sk1*-*Sk5*). For each frequency, one oscillation period is marked out for the skyrmions exhibiting significant oscillations; blue and red indicate a gyration phase difference of π.

Fig. S5.

FIG. S5. Benchmarking for breast cancer diagnosis using non-adaptive ANNs (A) Topology of the ANN used in direct data fusion. Layer 1 constitutes 30 neurons (blue circles) that take in biopsy data as direct input and four neurons (orange circles) that take in patient medical data also as direct input. (B) Topology of ANN used for baseline simulation. Layer 1 constitutes 30 neurons that take in biopsy data as direct input. (A & B) are created in Lucidchart [25].

Fig. S6.

FIG. S6. T-SKONE adapting to a dynamic environment. (A) Schematic of the human-machine interaction task. An intelligent agent receives spoken directions from a human being (red arrows), visually inspects a box (blue arrows) and decides whether to open it. (B) Flowchart of the decision-making process. (c-f) Plot of the oscillations of *Sk5* along *y*. The combinations of direct and control inputs are (‘0’, ‘0’) in (C), (‘0’, ‘1’) in (D), (‘1’, ‘0’) in (E) and (‘1’, ‘1’) in (F), respectively. Direct inputs ‘0’ and ‘1’ encode a sinusoidal excitation of frequency 1.22 GHz and 1.85 GHz, respectively. Control inputs ‘0’ and ‘1’ lead to T-SKONE in Configuration I and II, respectively. (A,B) are created in Lucidchart [25].

Fig. S7.

FIG. S7. Examination of the context input sequence. The figure plots the accuracy performance of the context-aware network for different sequences in which the context data is input to Layer 1. In Arrangement 1, the context data is input in the sequence (𝜶_1_, 𝜶_2_, 𝜶_3_, 𝜶_4_) and fed into (7, 7, 8, 8) neurons of Layer 1. In Arrangement *i* (*i*>1) the input sequence is altered such that is 𝜶_i_ swapped with 𝜶_1_. Therefore, in Arrangement 2, the context input sequence is (𝜶_2_, 𝜶_1_, 𝜶_3_, 𝜶_4_). The simulations show that the network performance depends on the choice of context sequence.

Fig. S8.

FIG. S8. Comparision of T-SKONE performance with state-of-the-art. The figure compares the performance of T-SKONE with state-of-the-art non-adaptive neurons for the breast cancer diagnosis task. The software plot (purple) shows the accuracy results for the direct data fusion method using a software perceptron and the context plot (green) shows the context-aware diagnosis method using a network of T-SKONEs. Both networks have 34 input neurons, 2 output neurons and 68 synapses.

Fig. S9.

FIG. S9. Schematic of two hidden neurons considered for Layer-wise Iterative Pruning. The figure shows a section of the input layer (Layer 1), the hidden layer (Layer 2), the output layer (Layer 3) and elements of the weight matrix W^1^ (corresponding to Layer 1) and W^2^ (corresponding to Layer 2). An element $W_{ij}^{1}$corresponds to the weight associated with the i^th^ neuron of Layer 1 and j^th^ neuron of Layer 2. The pruning algorithm compares the rows of the matrix W^1^ and eliminates redundant neurons.

Fig. S10.

FIG. S10. Demonstration of stability and repeatability of T-SKONE performance. (A-C) Plots of the amplitudes of oscillation for skyrmion 4 along *x* for an external magnetic field of 0.80 GHz, for Configuration I (blue circles) and Configuration II (magenta triangles). (A) Plot for device performance including thermal fluctuations. (B & C) Plots for device performance with variations in DMI and saturation magnetization, respectively.

Fig. S11.

FIG. S11. Demonstration of the adaptability of T-SKONE with time. (A) plots the variation of output amplitudes of oscillation of skyrmion 2 when T-SKONE is switched between Configurations I & II (switching marked by red line) under a constant oscillating magnetic field input. The neuronal output adapts to its new state within 20 ns. (B & C) plot the decay of output amplitudes of skyrmion 4 along x after an external magnetic field is switched off (switching marked by red lines). The neuronal output decays to 50% of its original value in 10 ns and reaches a negligible value by 30 ns.

Fig. S12.

FIG. S12. Demonstration of T-SKONE performance for large machine learning datasets. The figure demonstrates the performance of the adaptive ANN (green) for the widely used MNIST digit recognition task and compares it to a direct-fusion (orange) and a baseline network (blue). The baseline network uses 794 neurons comprising 28*28 input neurons and 10 output neurons while the adaptive and direct-fusion networks have an additional input neuron. The MNIST calculation uses 60,000 images in the training set and 10,000 images in the validation set. The adaptive ANN slightly outperforms the direct-fusion network, reaching the same final accuracy while learning faster.

**Table S1.**

Table S1. Contextual data used for diagnosis of breast cancer. The table lists the values used for the four attributes that serve as contextual variables in the diagnosis.

| Attribute (𝛂_i_) | Number of cancer patients with *𝛂_i_* = ‘True’ | Number of cancer patients with *𝛂_i_* = ‘False’ | Number of non-cancer patients with *𝛂_i_* = ‘True’ | Number of non-cancer patients with *𝛂_i_ = ‘False’* |
| --- | --- | --- | --- | --- |
| Sex is Female | 211 | 1 | 211 | 146 |
| Age >= 50 years | 160 | 52 | 13 | 344 |
| BMI > 30 | 147 | 65 | 98 | 259 |
| Alcohol intake is high | 181 | 31 | 272 | 85 |

# References

| [1] | Q. Shao, Y. Liu, G. Yu, S. Kim, X. Che, C. Tang, Q. He, Y. Tserkovnyak, J. Shi and K. Wang, "Topological Hall effect at above room temperature in heterostructures composed of a magnetic insulator and a heavy metal," *Nature Electronics,* vol. 2, no. 5, pp. 182-186., 2019. |
| --- | --- |
| [2] | O. Ciubotariu, A. Semisalova, K. Lenz and M. Albrecht, "Strain-induced perpendicular magnetic anisotropy and Gilbert damping of Tm 3 Fe 5 O 12 thin films," *Scientific reports,* vol. 9, no. 1, pp. 1-8, 2019. |
| [3] | J. Kim, J. Yang, Y.-J. Cho, B. Kim and S.-K. Kim , "Coupled gyration modes in onedimensional skyrmion arrays in thin-film nanostrips as new type of information carrier," *Scientific Reports,* vol. 7, p. 45185, 2017. |
| [4] | P. Lichtsteiner, T. Delbruck and J. Kramer, "Improved ON/OFF temporally differentiating address-event imager," in *Proceedings of the 11th IEEE International Conference on Electronics, Circuits and Systems (ICECS)*, Tel-Aviv, 2004. |
| [5] | A. Vanarse, A. Osseiran and A. Rassau, "A Review of Current Neuromorphic Approaches for Vision, Auditory, and Olfactory Sensors," *Frontiers in Neuroscience,* vol. 10, p. 115, 2016. |
| [6] | G. Yu, P. Upadhyaya, Q. Shao, H. Wu, G. Yin, X. Li, C. He, W. Jiang, X. Han, P. Amiri and K. Wang, "Room-temperature skyrmion shift device for memory application," *Nano letters,* vol. 17, no. 1, pp. 261-268, 2017. |
| [7] | S. Lin, C. Batista and A. Saxena, "Internal modes of a skyrmion in the ferromagnetic state of chiral magnets," *Physical Review B,* vol. 89, no. 2, p. 024415, 2014. |
| [8] | J. Kim, J. Yang, Y. Cho, B. Kim and S. Kim, "Coupled gyration modes in one-dimensional skyrmion arrays in thin-film nanostrips as new type of information carrier," *Scientific reports,* vol. 7, p. 45185, 2017. |
| [9] | O. L. Mangasarian and W. H. Wolberg, "Cancer diagnosis via linear programming," *SIAM News,* vol. 23, no. 5, pp. 1, 18, 1990. |
| [10] | C. E. Floyd, et al., "Prediction of Breast Cancer Malignancy Using an Artificial Neural Network," *Cancer,* vol. 74, no. 11, p. 2944, 1994. |
| [11] | Y.-S. Sun, et al., "Risk Factors and Preventions of Breast Cancer," *International Journal of Biological Sciences,* vol. 13, no. 11, pp. 1387-1397, 2017. |
| [12] | A. McGuire, J. A. L. Brown, C. Malone, R. McLaughlin and M. J. Kerin, "Effects of Age on the Detection and Management of Breast Cancer," *Cancers,* vol. 7, no. 2, p. 908, 2015. |
| [13] | N. Khan, F. Afaq and H. Mukhtar, "Lifestyle as risk factor for cancer: Evidence from human studies," *Cancer Letters,* vol. 293, no. 2, pp. 133 - 143, 2010. |
| [14] | J. Jang, J. Lee, S. Woo, D. J. Sly, L. J. Campbell, J.-H. Cho, S. J. O’Leary, M.-H. Park, S. Han, J.-W. Choi, J. H. Jang and H. Choi, "A microelectromechanical system artificial basilar membrane based on a piezoelectric cantilever array and its characterization using an animal model," *Scientific Reports,* vol. 5, p. 12447, 2015. |
| [15] | [Online]. Available: https://stats.stackexchange.com/questions/237169/why-are-non-zero-centered-activation-functions-a-problem-in-backpropagation. |
| [16] | W. Chu, "Layer-wise Iterative Pruning for Neural Network," in *ANU Bio-inspired Computing conference (ABCs)*, Canberra, 2018. |
| [17] | S. Moro, P. Cortez and P. Rita, "A data-driven approach to predict the success of bank telemarkeing," *Decision Supporting System,* vol. 62, pp. 22-31, 2014. |
| [18] | W. F. Brown Jr., "Thermal fluctuations of a single-domain particle," *Phys. Rev.,* vol. 130, no. 1677, 1963. |
| [19] | H. H. Zhu et al., "Space-efficient optical computing with an integrated chip diffractive neural network," *Nature Communications,* vol. 13, p. 1044, 2022. |
| [20] | Z. Wang et al., "In situ training of feed-forward and recurrent convolutional memristor networks," *Nature Machine Intelligence,* vol. 1, p. 434–442, 2019. |
| [21] | S. Choi et al., "SiGe epitaxial memory for neuromorphic computing with reproducible high performance based on engineered dislocations," *Nature Materials,* vol. 17, p. pages 335–340, 2018. |
| [22] | D. Brunner and D. Psaltis, "Competitive photonic neural networks," *Nature Photonics,* vol. 15, p. 323–324 , 2021. |
| [23] | P. Yao et al., "Fully hardware-implemented memristor convolutional neural network," *Nature,* vol. 577, p. 641–646, 2020. |
| [24] | Y. LeCun, L. Bottou, Y. Bengio and P. Haffner, "Gradient-based learning applied to document recognition," *Proceedings of the IEEE,* vol. 86, no. 11, pp. 2278-2323, 1998. |
| [25] | [Online]. Available: https://www.lucidchart.com/. |
